# Septohippocampal transmission from parvalbumin-positive neurons features rapid recovery from synaptic depression

**DOI:** 10.1038/s41598-020-80245-w

**Published:** 2021-01-22

**Authors:** Feng Yi, Tavita Garrett, Karl Deisseroth, Heikki Haario, Emily Stone, J. Josh Lawrence

**Affiliations:** 1 Department of Biomedical and Pharmaceutical Sciences, The University of Montana, Missoula, MT 59812 USA; 2 Vollum Institute Neuroscience Graduate Program, Oregon Health and Science University, Portland, OR 97239 USA; 3 Department of Psychiatry and Behavioral Sciences, Department of Bioengineering, and Howard Hughes Medical Institute, Stanford University, Stanford, CA 94305 USA; 4 Department of Mathematics and Physics, Lappeenranta University of Technology, Lappeenranta, Finland; 5 Department of Mathematical Sciences, The University of Montana, Missoula, MT 59812 USA; 6 Department of Pharmacology and Neuroscience, Garrison Institute on Aging, and Center for Excellence in Translational Neuroscience and Therapeutics, Texas Tech University Health Sciences Center, Lubbock, TX 79430 USA

**Keywords:** Hippocampus, Synaptic plasticity, Biophysical models, Neural circuits, Inhibition, Intrinsic excitability, Synaptic transmission

## Abstract

Parvalbumin-containing projection neurons of the medial-septum-diagonal band of Broca ($\hbox {PV}_{\text{MS-DBB}}$) are essential for hippocampal rhythms and learning operations yet are poorly understood at cellular and synaptic levels. We combined electrophysiological, optogenetic, and modeling approaches to investigate $\hbox {PV}_{\text{MS-DBB}}$ neuronal properties. $\hbox {PV}_{\text{MS-DBB}}$ neurons had intrinsic membrane properties distinct from acetylcholine- and somatostatin-containing MS-DBB subtypes. Viral expression of the fast-kinetic channelrhodopsin ChETA-YFP elicited action potentials to brief (1–2 ms) 470 nm light pulses. To investigate $\hbox {PV}_{\text{MS-DBB}}$ transmission, light pulses at 5–50 Hz frequencies generated trains of inhibitory postsynaptic currents (IPSCs) in CA1 stratum oriens interneurons. Using a similar approach, optogenetic activation of local hippocampal PV ($\hbox {PV}_{\text{HC}}$) neurons generated trains of $\hbox {PV}_{\text{HC}}$-mediated IPSCs in CA1 pyramidal neurons. Both synapse types exhibited short-term depression (STD) of IPSCs. However, relative to $\hbox {PV}_{\text{HC}}$ synapses, $\hbox {PV}_{\text{MS-DBB}}$ synapses possessed lower initial release probability, transiently resisted STD at gamma (20–50 Hz) frequencies, and recovered more rapidly from synaptic depression. Experimentally-constrained mathematical synapse models explored mechanistic differences. Relative to the $\hbox {PV}_{\text{HC}}$ model, the $\hbox {PV}_{\text{MS-DBB}}$ model exhibited higher sensitivity to calcium accumulation, permitting a faster rate of calcium-dependent recovery from STD. In conclusion, resistance of $\hbox {PV}_{\text{MS-DBB}}$ synapses to STD during short gamma bursts enables robust long-range GABAergic transmission from MS-DBB to hippocampus.

## Introduction

Parvalbumin-positive projection neurons from the medial septum-band of Broca ($\hbox {PV}_{\text{MS-DBB}}$) drive hippocampal theta oscillations^1–4^ and play important roles in sensory perception^5^, fear learning^6^, memory retrieval^7^, memory consolidation^8^, spatial working memory^9^, and neurogenesis^10^. During firing in vivo, $\hbox {PV}_{\text{MS-DBB}}$ neurons exhibit an intriguing pattern of 2–6 action potentials at gamma frequency, nested in a theta rhythm^1,11,12^. $\hbox {PV}_{\text{MS-DBB}}$ projection neurons exclusively target hippocampal interneurons in all subregions of the hippocampus^13–17^. The synaptic drive of this inhibitory GABAergic input forms a powerful disinhibition circuit, whereby $\hbox {PV}_{\text{MS-DBB}}$ neurons rhythmically inhibit firing of local hippocampal (HC) interneurons, which in turn rhythmically disinhibit GABAergic transmission onto principal cells^18,19^. This synchronization mechanism is a crucial driver of the “atropine-insensitive” hippocampal theta rhythm^1–3,19–23^.

Septohippocampal $\hbox {PV}_{\text{MS-DBB}}$ terminals are larger and more proximally located than local hippocampal interneuron terminals^14,16,24^, suggesting functional differences. Previous studies have used a minimal extracellular stimulation technique to stimulate septohippocampal GABAergic axons onto hippocampal oriens-lacunosum moleculare (O-LM) interneurons and found reduced paired-pulse depression (PPD) and increased coefficient of variation (CV) relative to GABAergic input from local VIP interneurons^25,26^. These observations suggest synaptic differences in short-term plasticity between septohippocampal and local GABAergic synapses. A subsequent study using optogenetic stimulation found that MS-DBB GABAergic transmission undergoes short-term depression (STD) that is sensitive to $\hbox {GABA}_{\text{B}}$ receptor antagonism^27^. While these studies have provided general knowledge of septohippocampal GABAergic transmission dynamics, the septohippocampal GABAergic projection is comprised of several GABAergic neuron subtypes, which include not only $\hbox {PV}_{\text{MS-DBB}}$ projection neurons but also cholinergic projection ($\hbox {ChAT}_{\text{MS-DBB}}$) neurons, which have recently been demonstrated to co-transmit both acetylcholine and GABA^28,29^. An additional somatostatin-positive MS-DBB cell type ($\hbox {SOM}_{\text{MS-DBB}}$), which we describe here, may also project to hippocampal subpopulations.

$\hbox {PV}_{\text{MS-DBB}}$ neurons have emerged as major contributors to behaviorally-relevant hippocampal network rhythms and learning operations^1,5,6,8,9^. What is missing is a precise understanding of the synaptic dynamics of $\hbox {PV}_{\text{MS-DBB}}$-mediated transmission onto specific interneuron targets. This knowledge is needed to mechanistically link the activation of neurochemically-specific MS-DBB neuron subtypes to the generation of hippocampal theta and gamma rhythms. Here, using optogenetic stimulation of $\hbox {PV}_{\text{MS-DBB}}$ neurons, we provide a comprehensive quantitative description of $\hbox {PV}_{\text{MS-DBB}}$-mediated transmission onto hippocampal stratum oriens interneurons across a dynamic range of in vivo firing frequencies^1,30,31^. We find that $\hbox {PV}_{\text{MS-DBB}}$-mediated transmission reaches steady-state short-term depression (STD) at both theta and gamma frequencies. However, compared to transmission from local $\hbox {PV}_{\text{HC}}$ synapses onto CA1 pyramidal cells (PCs), $\hbox {PV}_{\text{MS-DBB}}$-mediated transmission resists STD initially during gamma (20–50 Hz) frequency spike trains. Using mathematical synapse models, we find that kinetic differences in STD can be explained by different presynaptic calcium sensitivities that determine differential rates of calcium-dependent recovery from depression between $\hbox {PV}_{\text{MS-DBB}}$ and local $\hbox {PV}_{\text{HC}}$ synapses. We conclude that resistance to STD is a key feature of $\hbox {PV}_{\text{MS-DBB}}$-mediated transmission, facilitating robust information transfer from MS-DBB to hippocampus.

## Methods

### Ethics statement

All experimental procedures were approved by the University of Montana’s Institutional Animal Care and Use Committee (IACUC; approved Animal Use Protocols 026-11, 035-13, and 017-14) and performed in accordance with University of Montana’s IACUC guidelines and regulations.

### Transgenic mice

PV-CRE (stock # 008069; Jackson Labs, Bar Harbor, ME)^32^, SOM-CRE (Jackson Laboratories, stock # 013044)^33^, and ChAT-CRE (GM24 founder line, 017269-UCD, Mutant Mouse Regional Resource Center, Davis, CA)^34^ were maintained similarly to previous studies^35^. To visualize yellow fluorescent protein (YFP) in CRE-containing MS-DBB neurons, homozygous CRE mouse lines were crossed with a homozygous Rosa26EYFP reporter line^36,37^ (stock # 007920; Jackson Laboratories) to generate F1 heterozygous PV-$\hbox {CRE}^{{+/-}}$:Rosa26$\hbox {EYFP}^{{+/-}}$ (PV-Rosa), SOM-$\hbox {CRE}^{{+/-}}$:Rosa26$\hbox {EYFP}^{{+/-}}$ (SOM-Rosa), or ChAT-$\hbox {CRE}^{{+/-}}$:Rosa26$\hbox {EYFP}^{{+/-}}$ (ChAT-Rosa) mice, respectively. After weaning, gender-specific littermates were socially housed in groups of 2–5 per cage until use.

### Acute slice preparation

For electrophysiological characterization of $\hbox {PV}_{\text{MS-DBB}}$, $\hbox {SOM}_{\text{MS-DBB}}$ and $\hbox {ChAT}_{\text{MS-DBB}}$ neurons, acute coronal MS-DBB and/or transverse hippocampal slices (300 μm thickness) were obtained from 3–7 week-old mice^38^. Mice, continuously anesthetized with 4% isofluorane, were transcardially perfused with oxygenated, ice-cold, sucrose-based artificial cerebrospinal fluid (SB-ACSF) containing (in mM): 80 NaCl, 2.5 KCl, 24 $\hbox {NaHCO}_{\text{3}}$, 0.5 $\hbox {CaCl}_{\text{2}}$, 4 $\hbox {MgCl}_{\text{2}}$, 1.25 $\hbox {NaH}_{\text{2}} \hbox {PO}_{\text{4}}$, 25 glucose, 75 sucrose, 1 ascorbic acid, and 3 sodium pyruvate, saturated with 95% $\hbox {O}_{\text{2}}$–5% $\hbox {CO}_{\text{2}}$, pH 7.4. Following decapitation, heads were submerged in ice-cold SB-ACSF for 1–2 min. After the brain was exposed, a tissue block was made by isolating the cortex from the olfactory bulbs and cerebellum. A small lab weighing spatula (Fisher scientific, Cat No. NC1862870, MedicusHealth 150 mm size) was used to gently pry up and transfer the tissue block to a petri dish containing SB-ACSF. For optogenetics experiments, coronal sections of MS-DBB and/or transverse sections of hippocampus were cut simultaneously on a Vibratome 1200S (Leica Microsystems, Bannockburn, IL). The bottom surface of the slice, as it was cut free on the vibratome, was gently transferred via a open-tipped plastic transfer pipette and flipped, bottom-side up, into an incubation chamber containing SB-ACSF at 36 $^\circ$C. To minimize vertical vibration of the blade^38,39^, the Leica Vibrocheck device was employed prior to each use. Acute slices were incubated for at least 30 min prior to use.

### Stereotaxic injection of adeno-associated virus (AAV) into PV-CRE mice

AAV5 EF1a-DIO-ChR2(E123A)-EYFP (ChETA-YFP; University of North Carolina Vector Core, Chapel Hill, NC)^40^ was injected bilaterally (3 μL total; 1.5 μL/hemisphere; rate: 0.25 μL/min) into the MS-DBB (coordinates AP 1.1, ML 0.2, DV − 4.1 mm from bregma) or hippocampus (coordinates AP − 2.8, ML 3.3, DV − 2.3 mm from bregma) of 2.5–6 month old PV-CRE mice (20–30 g), using a previously described procedure^35^. To ensure adequate ChETA-YFP density, immunocytochemical or electrophysiological experiments were performed at least 40 days after survival surgery. MS-DBB slices were screened for ChETA-YFP prior to optogenetics experiments in hippocampal slices. For each mouse, only one brain region, MS-DBB or hippocampus, was injected with ChETA-YFP AAV.

### Whole cell recordings

Glass pipettes (cat # TW150F-3, World Precision Instruments; Sarasota, FL) were fabricated using a two-step PC-10 vertical puller (Narishige, East Meadow, NY). Pipette resistances (2.5–4.5 $M\Omega$) were used for whole-cell recordings. Whole-cell data, obtained using a Multiclamp 700B amplifier (Molecular Devices, Union City, CA), were filtered at 4 kHz, digitized at 20 kHz (Digidata 1440A, Molecular Devices), and acquired using Axograph X (Axograph Scientific, Sydney, Australia) running on a PC. ACSF (in mM: 125 NaCl, 2.5 KCl, 25 $\hbox {NaHCO}_{\text{3}}$, 2 $\hbox {CaCl}_{\text{2}}$, 1 $\hbox {MgCl}_{\text{2}}$, 1.25 $\hbox {NaH}_{\text{2}} \hbox {PO}_{\text{4}}$, and 20 glucose, saturated with 95% $\hbox {O}_{\text{2}}$/5% $\hbox {CO}_{\text{2}}$, pH 7.4) was heated to 34–35 $^\circ$C with HPT-2 (Scientifica, East Sussex, UK) or SH-27B/TC-324B (Warner, Hamden, CT) inline solution heaters. Whole cell mode was achieved once cell-attached resistance reached at least 1 $G\Omega$. In current clamp recordings (Table S1), initial access resistance ($\hbox {R}_{\text{a}}$) did not differ significantly among PV_MS-DBB_, SOM_MS-DBB_, and ChAT_MS-DBB_ cell types (p = 0.42, one-way ANOVA). Bridge balance was employed throughout current-clamp experiments. For current clamp recordings from acute MS-DBB slices, pipettes contained a potassium gluconate-based intracellular (IC) solution (in mM): 110 potassium gluconate, 40 KCl, 10 HEPES, 0.1 EGTA, 4 MgATP, 0.3 $\hbox {Na}_{\text{2}}$GTP, 10 phosphocreatine, and 0.2% biocytin, pH 7.2 and 295–305 mOsm. To maximize signal-to-noise and space clamp conditions in optogenetic experiments, postsynaptic pipettes contained a CsCl-based IC (in mM): 123 CsCl, 10 KCl, 30 HEPES, 5 EGTA, 4 MgATP, 0.3 $\hbox {Na}_{\text{2}}$GTP, 5 QX-314, 10 phosphocreatine and 0.2% biocytin (pH 7.2, and 295–305 mOsm). To isolate IPSCs, the extracellular solution contained the AMPA receptor antagonist DNQX (25 μM) and the NMDA receptor antagonist APV (50 μM). To isolate ChETA-induced photocurrents and action potentials, the $\hbox {GABA}_{\text{A}}$ receptor antagonist gabazine (5 μM) was added in addition to DNQX and APV.

### Visualization and photoactivation of EYFP-positive neurons

Live MS-DBB or hippocampal slices were adhered to poly-d-lysine-coated glass coverslips and visualized under a 63$\times$ objective using IR-Dodt contrast on an upright microscope (Axio Examiner, Carl Zeiss Microscopy, LLC) with a digital AxioCam FireWire camera. The microscope was integrated into either a Patch Pro 2000 (Scientifica, East Sussex, United Kingdom) or Infrapatch (Luigs and Neumann, Ratingen Germany). On the Patch Pro 2000, EYFP-positive neurons were visualized using 505 nm (LED4C11-SP, Thor Labs, Newton, NJ) or 470 nm (M470L2-C4, Thor Labs) LEDs controlled by 4-channel DC4100 or 1-channel DC2100 LED drivers (Thor Labs), respectively. On the Infrapatch, a Colibri LED Illumination system (Carl Zeiss Microscopy) containing 470 nm and 505 nm LEDs was used. Thor Labs controllers were connected to a SVB1 signal distribution box (Carl Zeiss Microscopy) that enabled LEDs to be controlled through AxioVision (Carl Zeiss Microscopy) for routine epifluorescence applications. For optogenetic stimulation on the Patch Pro 2000, a BNC cable connected the Digidata 1440A analog output to DC 2100 external trigger ports. On the Infrapatch, a BNC cable connected the Digidata 1440A TTL output to the Colibri external trigger port; light intensity was manually controlled with the Colibri. Both of these configurations enabled short (0.1–10 ms) 470 nm light pulses to be triggered within Axograph X (Sydney, Australia). For optogenetic stimulation of $\hbox {PV}_{\text{HC}}$ cells or $\hbox {PV}_{\text{MS-DBB}}$ fibers in acute hippocampal slices, the light intensity was adjusted to yield comparable IPSC amplitudes. The light intensity used for stimulation of $\hbox {PV}_{\text{HC}}$ cells was lower than that used for stimulation of $\hbox {PV}_{\text{MS-DBB}}$ fibers and terminals ($\hbox {p}<0.05$).

### Electrophysiological data analysis

Initial analysis of electrophysiological data was performed in Axograph X^35^. Immediately after whole-cell configuration was established, resting membrane potential ($\hbox {V}_{\text{m}}$) was determined by averaging over a 10–30 s duration window. Peak and steady state (SS) voltage responses were calculated, relative to the baseline voltage preceding the current step, by measuring the peak voltage response in the first 200 ms of a 1 s hyperpolarizing current step and measuring the SS response in a 200 ms window at the end of the current step, respectively. Input resistance ($\hbox {R}_{\text{in}}$) was calculated from the peak voltage response to a − 100 pA current step from − 60 mV. Sag was calculated as the ratio of SS to peak voltage to − 100 or − 200 pA current steps from − 60 mV. Membrane time constant ($\tau _{\text{m}}$) was obtained by a single exponential fit of the decay of voltage after a − 100 pA current injection. Cell capacitance ($\hbox {C}_{\text{m}}$) was calculated by dividing $\tau _{\text{m}}$ by the peak $\hbox {R}_{\text{in}}$. AP half width was determined from the time to reach 50% of peak to the time to reach 50% during repolarization. APs were detected using a derivative threshold by the Event Detection Plug-In Program in Axograph X using a threshold setting of 10 mV/ms. First AP half-width and height measures were obtained from the first event detected at the lowest current step amplitude. Single-cell AP half-width measurements were taken from a 1 s current step, binned in 100 ms increments, and averaged. Subsequently, bins were averaged across cell types. Due to the high release probability of the first IPSC amplitude in a train (P1), evoked IPSCs were unambiguously detected within a 1–2 ms time window after the 473 nm light pulse, which was readily distinguished from spontaneous IPSCs and background noise. Variance–mean analysis was performed similarly to previously described^41^. Briefly, 20-pulse trains at 5, 10, 20 and 50 Hz were delivered 7 times in semi-random order. The variance and mean for P2-P20 at 5, 10, 20 and 50 Hz were gathered and binned using 0.1 $\times$ P1 amplitude. The variance and mean of P1 together with the binned variance and mean of P2–P20 across all frequencies were fitted with a simple parabolic function:

1 $$\begin{aligned} \sigma ^{{2}} = (1 + \hbox {CV}^{{2}})qI - \hbox {I}^{{2}}/\hbox {N}_{\text{VM}} \end{aligned}$$

where $\sigma ^{{2}}$ is the IPSC variance, CV is the intrasite coefficient of variation (fixed at 0.3^42^), q is the quantal IPSC amplitude, and $\hbox {N}_{\text{VM}}$ is the number of functional release sites.

Release probability ($\hbox {p}_{\text{x}}$) is the averaged IPSC amplitude for x = 1–20 IPSC amplitudes within 5–50 Hz pulse trains. Maximum release probability ($\hbox {p}_{\text{max}}$) is calculated using the equation:

2 $$\begin{aligned} \hbox {p}_{\text{max}} = \hbox {p}_{\text{x}}/\hbox {N}_{\text{VM}}q \end{aligned}$$

### Anti-GFP and anti-PV immunocytochemistry

Heterozygous PV-$\hbox {CRE}^{{+/-}}$:Rosa26$\hbox {EYFP}^{{+/-}}$ mice were perfused with 60 ml of ice cold phosphate-buffered saline (PBS) containing (in mM: 137 NaCl, 2.7 KCl, 10 $\hbox {Na}_{\text{2}}$ $\hbox {HPO}_{\text{4}}$, 2 $\hbox {KH}_{\text{2}}$ $\hbox {PO}_{\text{4}}$, pH 7.4) followed by 48 ml of 4% paraformaldehyde (PFA; cat # 15714-S, Electron Microscopy Sciences Hatfield, PA). After post-fixation in 4% PFA overnight, hippocampal sections (50 μm thickness) were cut with a Vibratome VT1000 (Leica Microsystems, Inc.) in normal PBS. Sections were sequentially washed $3 \times 5$ min in PBS and incubated for 45 min in 0.3% Triton X-100 (in PBS; Fisher Scientific; cat # BP151-500) followed by 10% normal goat serum blocking solution (in PBS; cat # S-1000, Vector Labs, Burglingame, CA). Free-floating slices were incubated overnight at 4 $^\circ$C with chicken anti-GFP primary antibody (directed against YFP; cat# GFP-1020, 1:4000, Aves Labs, Inc.,Davis, CA, USA) and mouse anti-PV primary antibody (cat# P3088; Sigma-Aldrich, St Louis, MO, USA). Slices were then washed $3 \times 5$ min with PBS and incubated with goat anti-chicken Alexafluor 488 (1:500; cat# A-11039, Life Technologies, Grand Island, NY) and goat anti mouse Alexafluor 633 (1:500; cat# A-21050, Life Technologies). Finally, slices were stained with Neurotrace 435/455 (1:100; cat# N21479, Life Technologies) for 30 min and coverslipped with Vectashield Hardset Mounting Medium (cat# H-1400, Vector Labs). Images were acquired with a Fluoview FV-1000 confocal imaging system (Olympus, Center Valley, PA). PV-immunopositive cells and GFP positive cells were identified by their higher signal intensities compared to the background signal and confirmed by their co-localization with Neurotrace. Images were processed and counts were performed with ImageJ, Photoshop and Illustrator.

### Processing of biocytin-filled neurons and anti-GFP immunocytochemistry

Biocytin (0.2%; cat # B4261-100MG, Sigma-Aldrich) was included in the recording pipette for post-hoc morphological identification of recorded cells. After whole cell recording, an outside-outside out patch was obtained; slices were perfused for an additional 5–10 min to allow diffusion of biocytin into subcellular compartments. Slices were fixed overnight at 4 $^\circ$C in PBS containing 4% PFA, transferred to PBS, and kept for up to 2 weeks at 4 $^\circ$C. After permeabilization with 0.3% Triton X-100 in PBS for 2 h at room temperature, slices were incubated in PBS overnight at 16 $^\circ$C with Alexa 633-conjugated streptavidin (final concentration 1 μg/ml, cat # S-21375, Life Technologies) in PBS. Slices were cryoprotected overnight in PBS containing 30% sucrose, and then resectioned to 100–150 μm thickness using a sliding freezing microtome (HM430, Thermo Scientific, Waltham, MA). After $3 \times 5$ min washes with 0.3% Triton X-100 in PBS, slices were incubated with a blocking solution (0.05% Triton X-100, 0.1% sodium azide, 20% goat serum, in PBS) for 45 min and then incubated overnight in a carrier solution (0.05% Triton X-100, 0.1% sodium azide, 1% goat serum, in PBS) containing chicken anti-GFP primary (1:4000). Slices were incubated with the secondary antibody goat anti-chicken Alexafluor 488 (1:1000) for 1 hr. After staining with Neurotrace 435/455 (1:100) and mounting on gelatin-coated slides in Vectashield (Vector Labs, cat# H-1400), sections were imaged with a Fluoview FV-1000 confocal imaging system with a 25$\times$ objective (XLPL25XWMP, Olympus, Tokyo, Japan) and 60$\times$/1.45 oil objective. Confocal stacks were flat projected, rotated, and cropped in Image J^43^. To determine co-localization points of PV terminals and biocytin filled cells, acquired images (60$\times$, 800 dpi, 0.2 μm interval for z-axis) were deconvolved using Huygens 4.2 (SVI, Netherlands). Co-localization points were generated using the colocalization plug-in in Image J. Co-localization points were re-confirmed in 3D by verifying that colocalization points were apposed to ChETA-YFP positive boutons at the somatodendritic surface and a ChETA-YFP positive fiber passed through the colocalization point.

### Chemical reagents

DL-APV was obtained from R&D (R&D Systems, Minneapolis, MN). All other chemical reagents were purchased from Sigma-Aldrich (St. Louis, MO).

### Mathematical modeling of STD at $\hbox {PV}_{\text{MS-DBB}}$ and $\hbox {PV}_{\text{HC}}$ synapses

For modeling both the $\hbox {PV}_{\text{HC}}$ and $\hbox {PV}_{\text{MS-DBB}}$ STD data, we employed a two-dimensional discrete dynamical system originally developed for modeling CA1 PV basket cell (BC)-CA1 pyramidal cell transmission^41,44^. The models were implemented in Matlab (MathWorks, Natick, MA), and the Markov Chain Monte Carlo (MCMC) parameter estimation techniques were incorporated using the MCMC toolbox for Matlab available online^44^. For both $\hbox {PV}_{\text{MS-DBB}}$ and $\hbox {PV}_{\text{HC}}$ synapses, a nonlinear optimization was performed using average release proability ($\hbox {p}_{\text{r}}$) values from both the STD data set (4 frequencies $\times$ 20 trains/frequency = 80 values) and the recovery from paired pulse depression (PPD; P1 and P2 values from 6 interpulse intervals) data set. The parameter $\Delta$, defined as the ratio of the increase in [$\hbox {Ca}_{\text{2+}}$] after a stimulus $\delta _{\text{c}}$ divided by baseline [$\hbox {Ca}^{{2+}}$] level in the absence of a stimulus^41,44^, was assumed be invariant during STD ($\Delta$ fixed to 1) for both $\hbox {PV}_{\text{MS-DBB}}$ and $\hbox {PV}_{\text{HC}}$ synapses. The convergence criterion was set to 1.0e−4; optimization was achieved after 182–285 iterations. After optimization, MCMC analyses were performed with Markov chain lengths of 50,000–150,000. Final parameters are represented by the means and standard deviations of the sampled MCMC chains (Table 1).

**Table 1:** Model parameters.

Parameter	Description	SH	HC	$fold \Delta$
$\tau _{\text{Ca}}$	Decay constant for calcium (ms)	$27.7\pm 7.1$	$22.4 \pm 7.5$	1.2
$\hbox {P}_{\text{max}}$	Maximum $\hbox {p}_{\text{r}}$ (from VM analysis)	0.75	0.91	0.82
K	Half calcium concentration value for $\hbox {p}_{\text{r}}$ function	$0.69\pm 0.02$	$0.68 \pm 0.01$	1.01
$\hbox {k}_{\text{min}}$	Minimum rate of recovery of release sites	$9.0e^{-4} \pm 1.8e^{-4}$	$1.2e^{-3} \pm 1.5e^{-4}$	0.75
$\Delta k$	Absolute difference between $\hbox {k}_{\text{max}}$ and $\hbox {k}_{\text{min}}$	$0.017 \pm 0.004$	$9.4e^{-3} \pm 9.2e^{-4}$	1.8
$\hbox {K}_{\text{r}}$	Half calcium concentration value for rate of recovery function	$1.18 \pm 0.66$	$0.19 \pm 0.15$	6.2

### Statistical analysis

Statistical analyses were performed with Prism 6 (Graphpad Software, Inc., La Jolla, CA). Depending on the results of Shapiro–Wilk normality tests, paired *t* tests or Wilcoxon matched pairs signed rank tests were performed on parametric or nonparametric groups, respectively. For unpaired tests, unpaired *t* tests or Mann–Whitney tests were used. Parametric or non-parametric one-way ANOVA or two-way ANOVA was used for data with more than two groups, when appropriate. All data were presented as $means \pm SEM$ (n = number of recordings) with significance set at $\hbox {p} < 0.05$.

## Results

### $\hbox {PV}_{\text{MS-DBB}}$ neurons are distinct from $\hbox {SOM}_{\text{MS-DBB}}$ and $\hbox {ChAT}_{\text{MS-DBB}}$ neurons

We first sought to examine parvalbumin-positive MS-DBB ($\hbox {PV}_{\text{MS-DBB}}$) neurons relative to other neurochemically identified cell types within MS-DBB. $\hbox {PV}_{\text{MS-DBB}}$ neurons were visualized by crossing PV-CRE and Rosa26EYFP reporter lines^35^. As expected from reported anti-PV labeling in rat^18,19,45–47^ and viral expression in PV-CRE mice^48^, $\hbox {PV}_{\text{MS-DBB}}$ neurons were localized near the MS midline and were generally absent from the lateral septal nucleus (LSN; Fig. S1A). To compare $\hbox {PV}_{\text{MS-DBB}}$ neurons to other GABAergic cell types in MS-DBB, we crossed the same Rosa26EYFP reporter line with ChAT-CRE and SOM-CRE mice, enabling the visualization of acetylcholine- ($\hbox {ChAT}_{\text{MS-DBB}}$)^28^ and somatostatin ($\hbox {SOM}_{\text{MS-DBB}}$)-positive subtypes. In contrast to $\hbox {PV}_{\text{MS-DBB}}$ neurons, $\hbox {SOM}_{\text{MS-DBB}}$ (Fig. S1B) and $\hbox {ChAT}_{\text{MS-DBB}}$ (Fig. S1C) neurons were found more diffusely distributed in MS-DBB and LSN than $\hbox {PV}_{\text{MS-DBB}}$ cells. Consistent with our previous study^35^, hippocampal parvalbumin-positive ($\hbox {PV}_{\text{HC}}$) neurons exhibited anti-PV immunoreactivity (Fig. S2A–C). Similarly, $\hbox {PV}_{\text{MS-DBB}}$ neurons identified through YFP labeling were also PV-immunoreactive (Fig. S2D–F) and appeared to be distributed differently than YFP-positive $\hbox {SOM}_{\text{MS-DBB}}$ and $\hbox {ChAT}_{\text{MS-DBB}}$ populations (Fig. S1B and S1C). At higher magnification, anti-PV immunoreactivity appeared less intense and more variable in $\hbox {PV}_{\text{MS-DBB}}$ neurons (25.9%) than $\hbox {PV}_{\text{HC}}$ cells (85.0%), though anti-GFP labeling against YFP was intense for both $\hbox {PV}_{\text{HC}}$ and $\hbox {PV}_{\text{MS-DBB}}$ populations (Fig. S2). Overall, differences in the localization of $\hbox {PV}_{\text{MS-DBB}}$ and $\hbox {ChAT}_{\text{MS-DBB}}$ subtypes are consistent with previous studies in rat^15,18,49^ and mouse^50,51^. Due to their different localization within MS-DBB and little overlap with anti-PV neurons, $\hbox {SOM}_{\text{MS-DBB}}$ neurons appeared to be a distinct subpopulation compared to $\hbox {PV}_{\text{MS-DBB}}$ neurons (Fig. S1B4).

**Figure 1:**
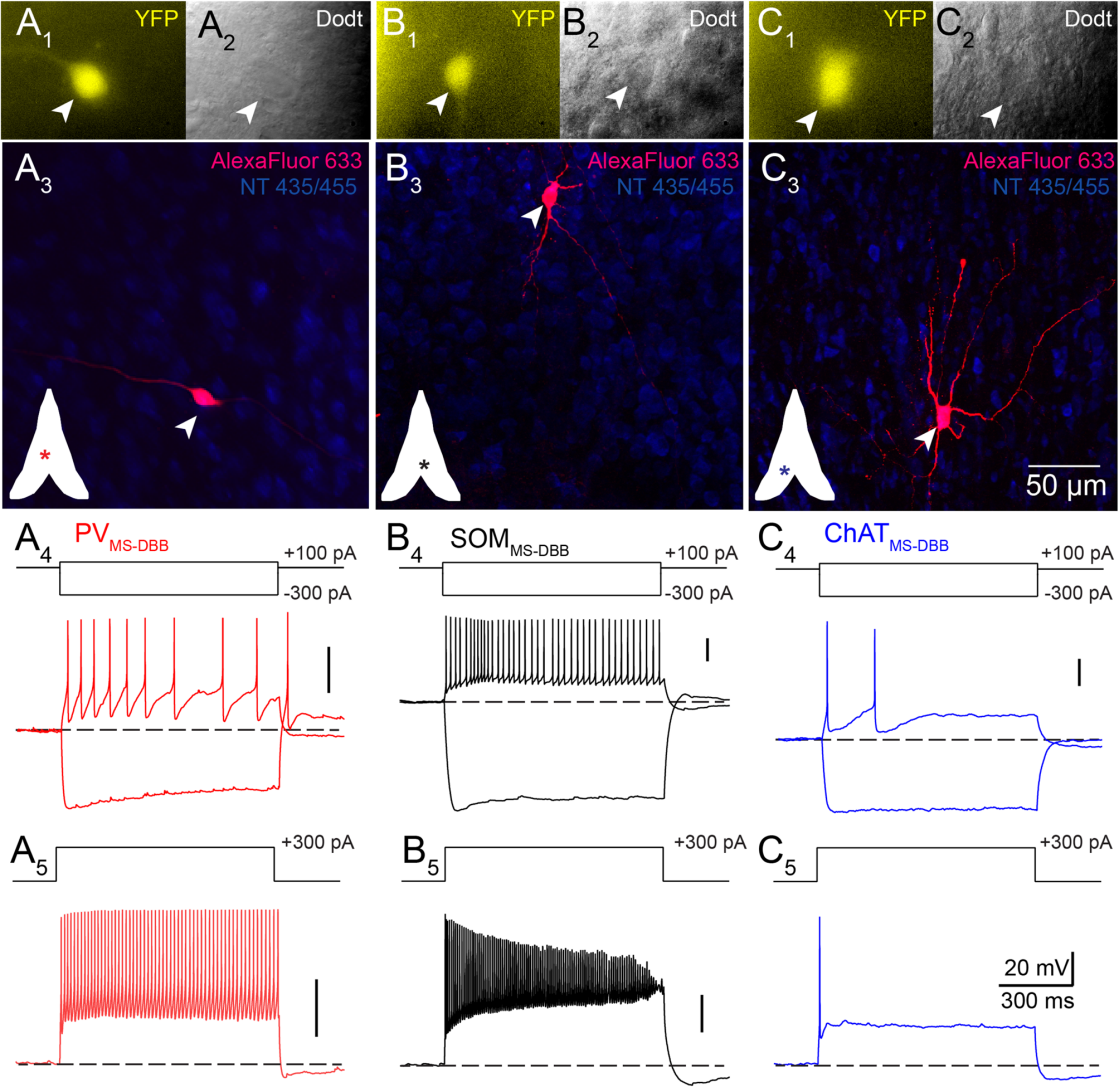
Passive and active properties of $\hbox {PV}_{\text{MS-DBB}}$, $\hbox {SOM}_{\text{MS-DBB}}$ and $\hbox {ChAT}_{\text{MS-DBB}}$ neurons. Images of (**A1**) live YFP, (**A2**) live IR-Dodt contrast, and (**A3**) flat-projected confocal stack of the representative $\hbox {PV}_{\text{MS-DBB}}$ neuron in (**A1**)–(**A2**) after post-hoc recovery of intracellular biocytin labeling. Inset depicts the cell location (asterisk) within MS-DBB. (**A4**) Voltage responses to −300 and +100 pA and (**A5**) +300 pA currents illustrate the fast-spiking phenotype of the $\hbox {PV}_{\text{MS-DBB}}$ and hyperpolarization-induced sag accompanied by a rebound spike. The $\hbox {PV}_{\text{MS-DBB}}$ neuron was maintained at −60 mV with the introduction of negative bias current. Representative (**B**) $\hbox {SOM}_{\text{MS-DBB}}$ and (**C**) $\hbox {ChAT}_{\text{MS-DBB}}$ neurons, formatted similarly to (**A**), exhibit differences in intrinsic properties.

**Figure 2:**
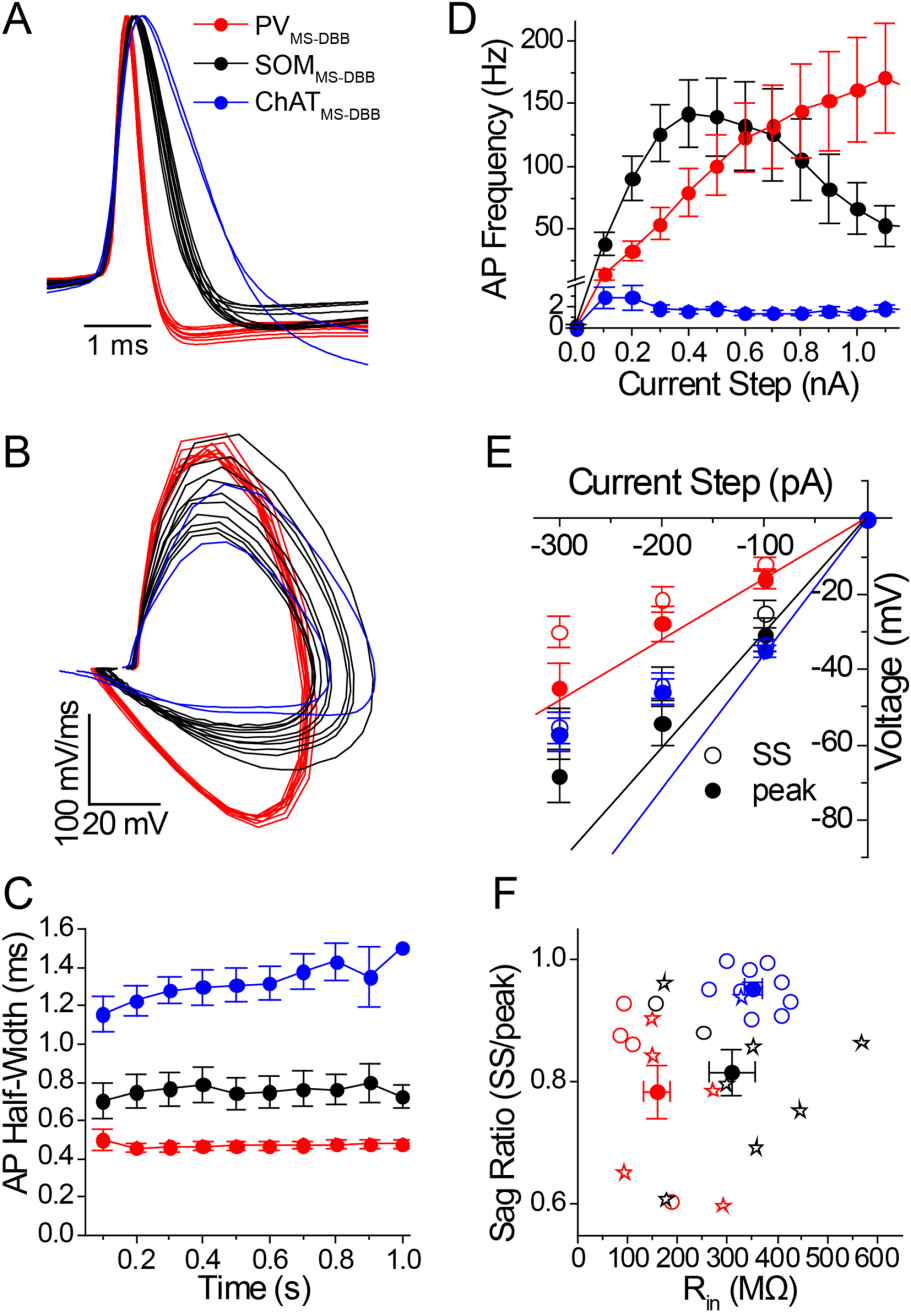
$\hbox {PV}_{\text{MS-DBB}}$ neurons are distinct from $\hbox {SOM}_{\text{MS-DBB}}$ and $\hbox {ChAT}_{\text{MS-DBB}}$ neurons. Overlaid are (**A**) normalized AP waveforms and (**B**) phase plots of APs of representative $\hbox {PV}_{\text{MS-DBB}}$ (red), $\hbox {SOM}_{\text{MS-DBB}}$ (black) and $\hbox {ChAT}_{\text{MS-DBB}}$ (blue) neurons. (**C**) AP half-width measurements during a 1 s long current step (+400 pA for $\hbox {PV}_{\text{MS-DBB}}$ and $\hbox {SOM}_{\text{MS-DBB}}$ cells; +100 pA for $\hbox {ChAT}_{\text{MS-DBB}}$ cells; 0.1 s bin). (**D**) Average AP frequencies generated in response to 1 s long, +100–1200 pA current steps from −60 mV. (**E**) Peak (closed circles) and steady-state (SS; open symbols) voltage responses to 1 s long −100 to −300 pA from −60 mV. To reveal rectification of voltage responses, Ohmic linear regression lines from zero to the peak voltage response at the −100 pA current step are shown. (**F**) A plot of sag ratio (SS/peak) vs. input resistance ($\hbox {R}_{\text{in}}$) distinguishes neurochemically distinct MS-DBB cell types. Open and closed symbols denote individual cells and population means, respectively. Open stars indicate neurons that exhibited hyperpolarization-induced rebound spikes.

To investigate whether $\hbox {PV}_{\text{MS-DBB}}$ neurons had intrinsic membrane properties distinct from $\hbox {SOM}_{\text{MS-DBB}}$ and $\hbox {ChAT}_{\text{MS-DBB}}$ subtypes, we performed whole-cell current clamp recordings from visually identified YFP-positive neurons. In a representative $\hbox {PV}_{\text{MS-DBB}}$ neuron, (Fig.1A1–3), depolarizing current steps of +100 pA (Fig.1A4) and +300 pA (Fig.1A5) amplitude evoked AP trains that were characteristic of a fast-spiking PV phenotype^52–54^. A −300 pA current step revealed a hyperpolarization-induced sag that was followed by a rebound spike. A similar current clamp protocol was performed on $\hbox {SOM}_{\text{MS-DBB}}$ and $\hbox {ChAT}_{\text{MS-DBB}}$ neurons. Upon injection of a +100 pA current step, high frequency firing was readily elicited in the $\hbox {SOM}_{\text{MS-DBB}}$ neuron (Fig. 1B4). However, during a +300 pA current step, action potential amplitudes decreased (Fig. 1A5). $\hbox {ChAT}_{\text{MS-DBB}}$ neurons elicited accommodating action potentials at +100 and +300 pA current steps (Fig. 1C4–5). An overlay of AP waveforms (Fig. 2A) and their respective phase plots (Fig. 2B) suggested that $\hbox {PV}_{\text{MS-DBB}}$, $\hbox {SOM}_{\text{MS-DBB}}$, and $\hbox {ChAT}_{\text{MS-DBB}}$ neurons were distinct cell populations. Within these populations, AP half-width was constant ($\hbox {p}>0.05$) over the 1 s current step. However, $\hbox {PV}_{\text{MS-DBB}}$ neurons exhibited a shorter AP half-width ($0.52 \pm 0.5$ ms) than $\hbox {ChAT}_{\text{MS-DBB}}$ neurons ($1.25 \pm 0.07$ ms, $\hbox {p}<0.0001$, Kruskal–Wallis test, followed by Dunn’s multiple comparisons test; Fig. 2C).

A quantitative examination of $\hbox {PV}_{\text{MS-DBB}}$, $\hbox {SOM}_{\text{MS-DBB}}$, and $\hbox {ChAT}_{\text{MS-DBB}}$ populations demonstrated differences in AP frequency across a range of 0.1–1.2 nA current steps (Fig. 2D). In the $\hbox {PV}_{\text{MS-DBB}}$ population (Fig. 2D, red), AP frequency increased linearly over 0.1–1.1 nA steps ($0.16 \pm 0.02$ Hz/pA, $\hbox {R}^{{2}}=0.33$, $\hbox {p}<0.0001$, n = 9). In the $\hbox {SOM}_{\text{MS-DBB}}$ population, AP frequency increased linearly during the first 0.1–0.4 nA steps ($0.37 \pm 0.06$ Hz/pA, $\hbox {R}^{{2}}=0.50$, $\hbox {p}<0.0001$; Fig. 2D, black), achieving a peak firing frequency of $142 \pm 27$ Hz at 400 pA, similar to $\hbox {PV}_{\text{MS-DBB}}$ neuron peak firing ($171 \pm 44$ Hz at 1100 pA) (p = 0.59, unpaired *t* test, t(13) = 0.56). However, in contrast to $\hbox {PV}_{\text{MS-DBB}}$ neurons, $\hbox {SOM}_{\text{MS-DBB}}$ neurons did not maintain a linear increase in AP frequency over 0.1–1.1 nA steps ($0.022 \pm 0.024$ Hz/pA, $\hbox {R}^{{2}}=0.008$, p = 0.36). Rather, a decrease in AP frequency across 0.4–1.1 nA current steps ($-\,0.14 \pm 0.04$ Hz/pA, $\hbox {R}^{{2}}=0.127$, p = 0.002, n = 9) was observed, resulting in a much lower AP frequency at 1.1 nA ($53 \pm 16$ Hz) relative to that of $\hbox {PV}_{\text{MS-DBB}}$ neurons (p = 0.036, unpaired *t* test; Fig. 2D, black), suggestive of sodium channel inactivation. For the $\hbox {ChAT}_{\text{MS-DBB}}$ population, a 1 s long, +100 pA current step generated low frequency APs ($2.8 \pm 0.9$ Hz), consistent with previous reports^55,56^. Across 0–1.1 nA steps, on average, $\hbox {ChAT}_{\text{MS-DBB}}$ cells did not exhibit a linear increase in AP frequency ($\hbox {R}^{{2}}=0.002$, p = 0.75, n = 5), with only 1–2 APs observed at large (0.3–1.1 nA) current steps (Fig. 2D, blue).

$\hbox {SOM}_{\text{MS-DBB}}$ neurons displayed higher peak input resistance ($\hbox {R}_{\text{in}}$; $308.7 \pm 46.1$ $M\Omega$) than $\hbox {PV}_{\text{MS-DBB}}$ ($158.0 \pm 26.0$ $M\Omega$, p = 0.005) neurons in response to a hyperpolarizing (−100 pA) current step (Fig. 2E,F; Table S1). Peak voltage deflections were significantly larger than steady-state voltage responses were observed in $\hbox {PV}_{\text{MS-DBB}}$ (paired *t* test, t(8) = 2.9, p = 0.02, n = 9), $\hbox {SOM}_{\text{MS-DBB}}$ (paired *t* test, t(8) = 4.6, p = 0.002, n = 9), and $\hbox {ChAT}_{\text{MS-DBB}}$ (Wilcoxon matched pairs signed rank test, W(10) = 55, p = 0.002, n = 10) neurons (Fig. 2F), indicating that all cell types displayed detectable sag upon hyperpolarization. Rebound spikes were observed in the majority of $\hbox {PV}_{\text{MS-DBB}}$ and $\hbox {SOM}_{\text{MS-DBB}}$ neurons (Table S1, 2F, open stars), consistent with HCN channel expression in $\hbox {PV}_{\text{MS-DBB}}$ and other MS-DBB GABAergic neurons^31,57^. Although hyperpolarization-induced sag was detected in $\hbox {ChAT}_{\text{MS-DBB}}$ neurons, this population exhibited a significantly lower sag ratio (SS/peak; $0.94 \pm 0.01$) than $\hbox {PV}_{\text{MS-DBB}}$ ($0.78 \pm 0.04$, p = 0.010) or $\hbox {SOM}_{\text{MS-DBB}}$ neurons ($0.82 \pm 0.04$, p = 0.032, Holm–Sidak’s multiple comparison’s test), suggestive of a relatively low density of HCN channels^31,57^. However, $\hbox {ChAT}_{\text{MS-DBB}}$ neurons^57^ exhibited strong steady-state inward rectification (p = 0.63, comparing voltage at −200 and −300 pA current steps), indicating the presence of inward rectifier potassium channels^58^. Despite major differences in AP firing (Fig. 2C,D), $\hbox {R}_{\text{in}}$ was not significantly different between $\hbox {SOM}_{\text{MS-DBB}}$ ($308.7 \pm 46.1$ $M\Omega$) and $\hbox {ChAT}_{\text{MS-DBB}}$ ($352 \pm 17$ $M\Omega$) neurons (p = 0.33, one-way ANOVA, followed by Holm–Sidak’s multiple comparisons test). $\hbox {V}_{\text{m}}$ (p = 0.21, one-way ANOVA), holding current at − 60 mV (p = 0.079, Kruskal–Wallis test), $\hbox {C}_{\text{m}}$ (p = 0.062, one-way ANOVA), and membrane time constant (p = 0.084, Kruskal–Wallis test) were not significantly different across $\hbox {PV}_{\text{MS-DBB}}$, $\hbox {SOM}_{\text{MS-DBB}}$ and $\hbox {ChAT}_{\text{MS-DBB}}$ populations (Table S1).

In summary, $\hbox {PV}_{\text{MS-DBB}}$ neurons were distinguished from $\hbox {SOM}_{\text{MS-DBB}}$ and $\hbox {ChAT}_{\text{MS-DBB}}$ neurons on the basis of $\hbox {R}_{\text{in}}$ and hyperpolarization-induced sag (Fig. 2F). Moreover, $\hbox {PV}_{\text{MS-DBB}}$ neurons exhibited shorter AP half-width than $\hbox {SOM}_{\text{MS-DBB}}$ and $\hbox {ChAT}_{\text{MS-DBB}}$ neurons (Fig. 2C).

### Expression of ChETA-YFP induces action potentials in $\hbox {PV}_{\text{MS-DBB}}$ and $\hbox {PV}_{\text{HC}}$ neurons

To investigate $\hbox {PV}_{\text{MS-DBB}}$-mediated synaptic transmission in the hippocampus, we stereotaxically injected ChETA-YFP AAV^40^ into the MS-DBB of PV-CRE mice. To compare with $\hbox {PV}_{\text{HC}}$-mediated transmission, we also made stereotaxic injections into the hippocampus. MS-DBB cells in live slices were confirmed to express ChETA-YFP (Fig. S3A,B). In the presence of ionotropic glutamate receptor (DNQX, APV) and $\hbox {GABA}_{\text{A}}$ receptor (gabazine) blockers, light flashes of 0.1–10 ms duration generated photocurrents with rapid onset and decay (Fig. S3D; $\tau _{\text{on}}=0.32 \pm 0.03$ ms, $\tau _{\text{off}}= 4.17 \pm 0.21$ ms for 0.1 ms light pulse (black), n = 3). Light pulses of 1–2 ms duration optimally evoked single APs with excellent temporal precision^40^, whereas longer duration (5–10 ms) light pulses triggered multiple APs (Fig. S3C). Suprathreshold spikes were elicited from 5 additional ChETA-expressing $\hbox {PV}_{\text{MS-DBB}}$ cells. $\hbox {PV}_{\text{MS-DBB}}$ neurons that expressed ChETA-YFP possessed fast spiking phenotypes ($184 \pm 9$ Hz at 700 pA; Fig. S3E,F) and brief AP half widths ($0.39 \pm 0.03$ ms; n = 6), which did not differ significantly from $\hbox {PV}_{\text{MS-DBB}}$ neurons labeled through the YFP reporter line (Fig. 2C; p = 0.26, U(17) = 13, Mann–Whitney U). This experimental design is similar to a previous report in which ChETA-YFP was used in vitro^59^. Similar to $\hbox {PV}_{\text{MS-DBB}}$ neurons, several representative $\hbox {PV}_{\text{HC}}$ neurons expressing ChETA-YFP had axonal arborizations targeting the PC layer, consistent with $\hbox {PV}_{\text{HC}}$ basket cells (Fig. S3G,H), which elicited APs upon optogenetic stimulation (Fig. S3K).

### Repetitive optogenetic stimulation of $\hbox {PV}_{\text{MS-DBB}}$ synapses induces STD of IPSCs onto CA1 stratum oriens interneurons

**Figure 3:**
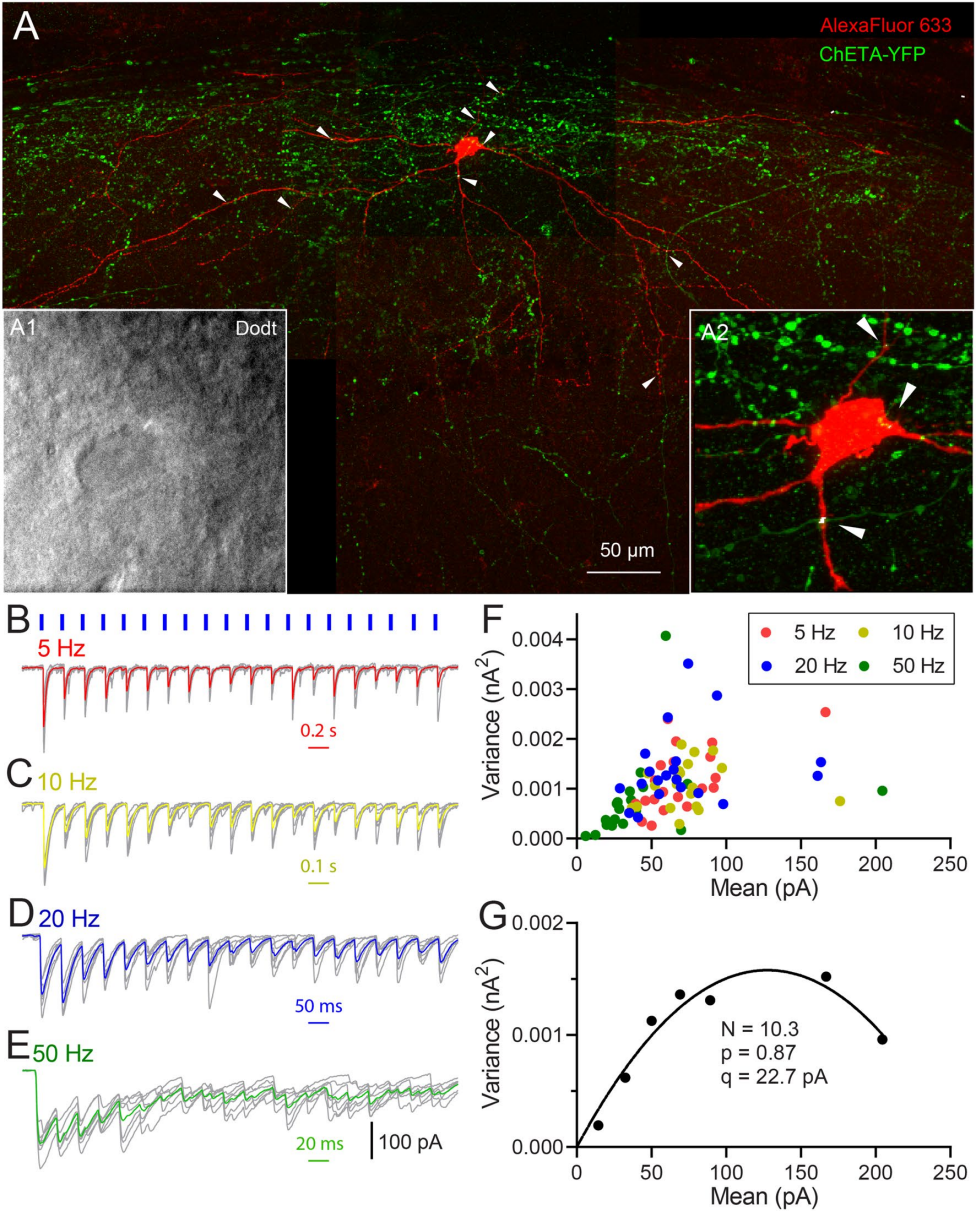
$\hbox {PV}_{\text{MS-DBB}}$ transmission onto a representative hippocampal CA1 stratum oriens interneuron. (**A**) Post-hoc recovery of a biocytin filled cell. Co-localization points of potential actual contact (red/greed overlap depicted in white) of ChETA-YFP axons (green) and biocytin-filled cell (red) are indicated by white arrows. (**A1**, inset) Live IR Dodt contrast of the recorded CA1 SO cell. (**A2**) magnified image of the cell body. (**B**–**E**) 5, 10, 20 and 50 Hz (20 pulses of 1 ms duration, depicted as blue short lines) 470 nm light pulse trains evoked IPSCs (raw traces,grey; average trace, colored). Each train was repeated 7 times. (**F**) The peak variance (V) and mean (M) was computed for each pulse in each train in (**B**–**E**). In (**F**), 20 VM points are shown for (red) 5 Hz, (yellow) 10 Hz, (blue) 20 Hz and (green) 50 Hz conditions, with each point representing the M and V of 7 individual IPSCs. VM points in F are binned in 20 pA increments (**G**). Assuming an intrasite quantal variance of 0.3^42^, the V–M relationship was fit by the parabolic function $\delta ^{2} = (1 + CV^{2})qI - I^{2}/N$, where $\delta ^{2}$ is the variance, I is the mean, N is the number of functional release sites, CV is the intrasite coefficient of variation, and q is the quantal amplitude.

**Figure 4:**
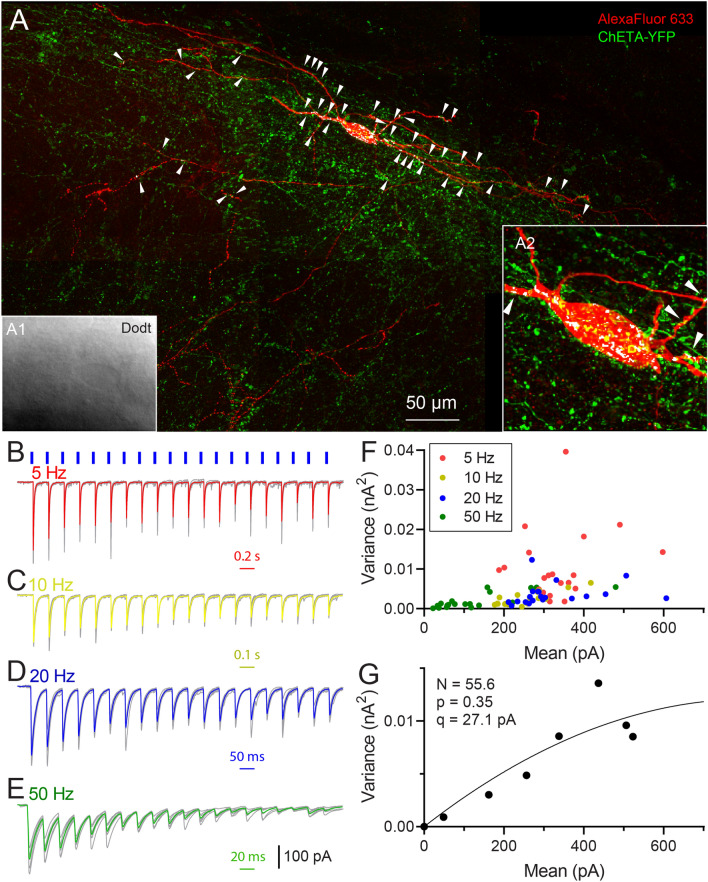
A second example of $\hbox {PV}_{\text{MS-DBB}}$ transmission onto a hippocampal CA1 stratum oriens interneuron. (**A**–**G**) are formatted similarly to Fig. 3. (**A**) A larger number of potential actual contacts were observed, which correlated with (**G**) a larger number of release sites extracted from VM analysis.

Hippocampal somatostatin (SOM)-containing interneurons of the CA1 stratum oriens (SO) are targeted by MS-DBB GABAergic neurons^16,60^ and receive sensory information from these neurons during behaviorally salient events^5^. Therefore, we focused our efforts on interneurons within the CA1 SO, which are enriched in this SOM interneuron population^54^. Optogenetically evoked IPSCs from $\hbox {PV}_{\text{MS-DBB}}$ afferents were detected in 20/52 (38.5%) of SO interneurons. A representative recording from a CA1 SO interneuron is shown in Fig. 3. Post-hoc recovery of the biocytin-filled SO interneuron (red), combined with visualization of ChETA-YFP synapses, enabled us to estimate the number of synaptic contacts (white dots, indicated by white arrows) onto the recorded SO interneuron (Fig. 3A). In response to trains of 20 light pulses (1 ms duration) at 5–50 Hz, inhibitory postsynaptic currents (IPSCs) were evoked with rapid onset and rise, consistent with monosynaptic $\hbox {PV}_{\text{MS-DBB}}$-mediated transmission (Fig. 3B–E). On average, initial IPSCs (P1) were large (415–601 pA) and robust. The second (P2) and subsequent (P3–P20) IPSCs were reduced in amplitude relative to P1, generally inducing paired pulse depression (PPD) and short-term depression (STD) at 5–50 Hz (Fig. 3B–E). Taking advantage of the stochastic nature of transmitter release, we employed variance–mean analysis^42,61,62^ to obtain the maximal $\hbox {p}_{\text{r}}$ ($\hbox {p}_{\text{max}}$), quantal amplitude (q), and number of functional release sites ($\hbox {N}_{\text{VM}}$) at $\hbox {PV}_{\text{MS-DBB}}$ synapses. Consistent with a high $\hbox {p}_{\text{max}}$, P1 amplitudes (Fig. 3B–E) were of high mean and low variance (Fig. 3F). Over the course of the trains, IPSC amplitudes ultimately clustered in a two-dimensional region of low variance and mean (Fig. 3F), suggesting depletion of the synaptic vesicle pool during the trains. After grand means and variances were fitted to Eq. (1) (see “Methods”), $\hbox {p}_{\text{max}}$ was 0.87, q was 22.7 pA, and $\hbox {N}_{\text{VM}}$ was 10.3 (Fig. 3G). The $\hbox {N}_{\text{VM}}$ was in close correspondence with potential actual contact (AC) points ($\hbox {N}_{\text{AC}}$, 9)(Fig. 3A, white arrows). In another SO interneuron, (Fig. 4A), $\hbox {N}_{\text{AC}}$ was 50 (Fig. 4D), consistent with the proportionally larger increase in calculated $\hbox {N}_{\text{VM}}$ of 55.6 (Fig. 4B–G). Of 12 SO interneurons with completed variance–mean analysis, 4 were successfully recovered. Among these 4 recovered cells, axonal arborizations of two cells (Figs. 3, 4, Fig. S4) were observed in the SLM layer, defining them as O-LM cells. Axonal arborizations were not detected in the other two cells, likely due to severing of the axon during the preparation of acute slices.

**Figure 5:**
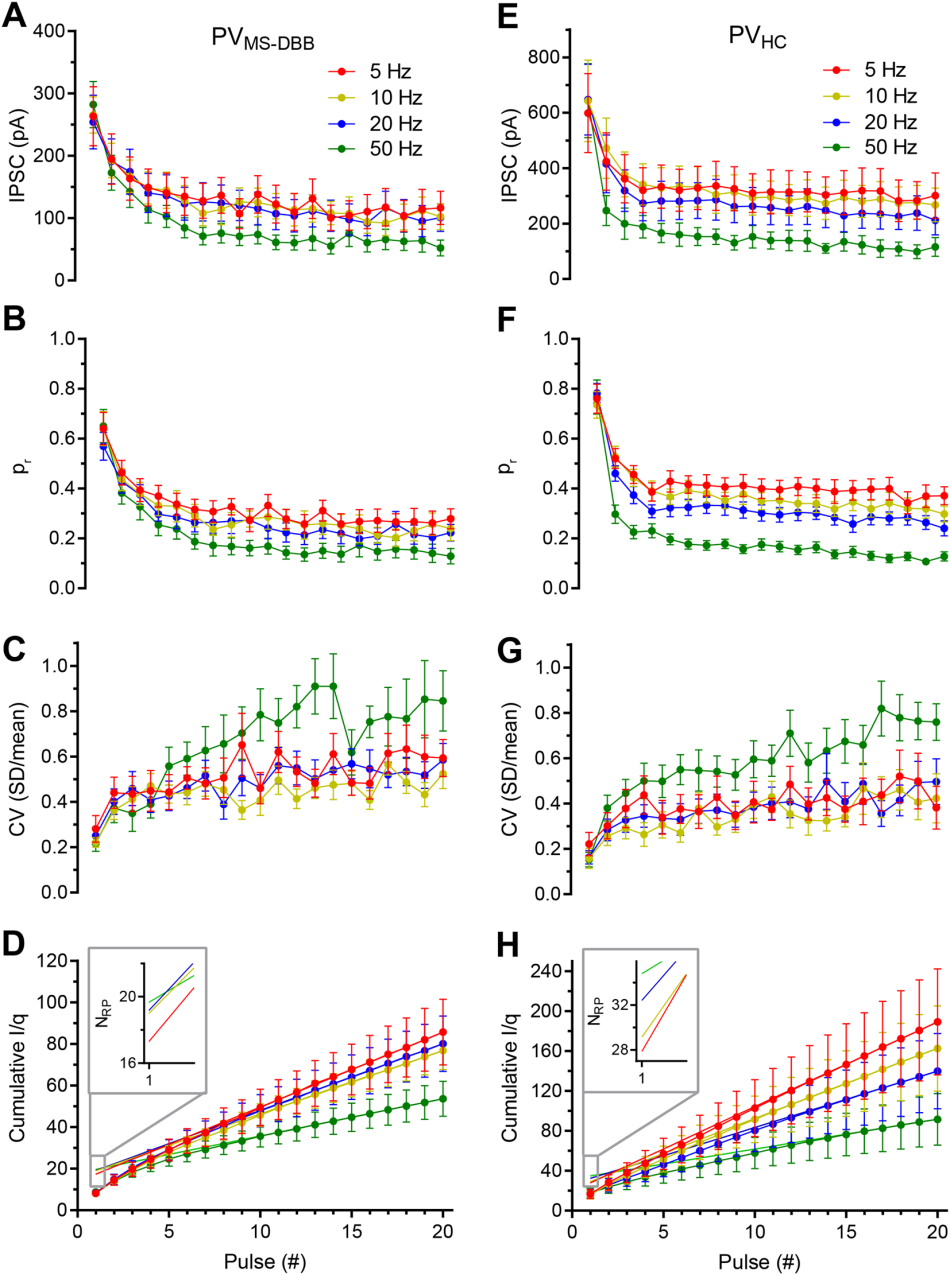
Frequency-dependence of $\hbox {PV}_{\text{MS-DBB}}$ and $\hbox {PV}_{\text{HC}}$-mediated transmission. (**A**) $\hbox {PV}_{\text{MS-DBB}}$ IPSC amplitude induced by optogenetic stimulation at 5 Hz (red), 10 Hz (yellow), 20 Hz (blue), and 50 Hz (green). (**B**) Release probability ($\hbox {p}_{\text{r}}$; IPSC amplitudes divided by Nq) across the P1–P20 pulse train at different frequencies. (**C**) Coefficient of variation (CV; SD/mean) for 5–50 Hz frequencies. (**D**) Cumulative ISPC amplitude (I) divided by quantal amplitude (q) for 5-50 Hz frequencies. Inset, linear fitting from last 5 pulse estimate the number of release sites at the intercept. (**E**–**H**) IPSC amplitude, $\hbox {p}_{\text{r}}$, CV, and cumulative I/q for $\hbox {PV}_{\text{HC}}$ neurons with the same format as (**A**–**D**).

As a population, optogenetic stimulation of $\hbox {PV}_{\text{MS-DBB}}$ afferents resulted in large P1 IPSC amplitudes (252–284 pA) in CA1 SO interneurons that were consistent in amplitude across all frequencies (repeated measures one-way ANOVA; p = 0.58, F(2.7,21.8) = 0.64, Fig. 5 A). $\hbox {PV}_{\text{MS-DBB}}$ synapses exhibited robust paired pulse depression (PPD) at all frequencies (5 Hz: p = 0.005, t(10) = 3.64, n = 11; 10 Hz: p = 0.002, t(10) = 4.23, n = 11; 20 Hz: p = 0.001, W(10) = - 66.0, n = 11; 50 Hz: p = 0.003, t(9) = 4.0, n = 10). Moreover, at each frequency, the coefficient of variation (CV) for P2 was larger than P1 ($\hbox {p}<0.05$, Wilcoxon matched-pairs signed rank test), suggesting a presynaptic mechanism. Similarly, STD was observed at all frequencies (Friedman test, $\hbox {p}<0.0001$, n = 11; Fig.5A), which was accompanied by a significant increase in CV at steady-state (P1 vs. the average of P11–20, $\hbox {p}<0.01$, n = 11, 11, 11, 10 for 5 Hz, 10 Hz, 20 Hz, 50 Hz respectively, Wilcoxon matched-pairs signed rank test; Fig. 5C). Therefore, both PPD and STD results are consistent with a presynaptic locus.

The high initial $\hbox {p}_{\text{r}}$ implied by the low variance of P1 permitted variance–mean (V–M) analysis (Fig.3G), allowing us to estimate the quantal amplitude (q: $36.4 \pm 6.0$ pA), maximum release probability ($\hbox {p}_{\text{max}}$: $0.64 \pm 0.07$) and number of functional release sites ($\hbox {N}_{\text{VM}}$: $15.4 \pm 4.0$) for a population of 12 postsynaptic SO interneurons (n = 12; Table S1). In four SO interneurons that allowed a complete post-hoc analyses of ChETA-positive terminals apposed to the somatodendritic domains of biocytin-filled cells, $\hbox {N}_{\text{AC}}$ was calculated to be $17.8 \pm 10.8$, which was not significantly different than the calculated $\hbox {N}_{\text{VM}}$ ($19.8 \pm 12$, Wilcoxon matched-pairs signed rank test, p = 0.38, n = 4) for these cells. Dividing P1–P20 IPSC amplitudes (Fig. 5A) by the maximum IPSC amplitude possible (N*q) enabled us to calculate $\hbox {p}_{\text{r}}$ during the train (Fig. 5B). As an independent measure of N, we calculated the number of functional release sites in the readily releasable pool ($\hbox {N}_{\text{RP}}$) by dividing the cumulative IPSC amplitude by q ($\hbox {N}_{\text{RP}}=17.2 \pm 2.3$, n = 12; Fig.5D). There was no significant difference in N, as measured by $\hbox {N}_{\text{AC}}$, $\hbox {N}_{\text{RP}}$ or $\hbox {N}_{\text{VM}}$ (Kruskal–Wallis test, p = 0.41, followed by Dunn’s multiple comparisons test). We conclude that repetitive optogenetic stimulation of $\hbox {PV}_{\text{MS-DBB}}$ synapses at 5–50 Hz evokes large, depressing IPSCs onto CA1 SO interneurons.

### Repetitive optogenetic stimulation of $\hbox {PV}_{\text{HC}}$ synapses induces STD of IPSCs onto CA1 PCs

**Figure 6:**
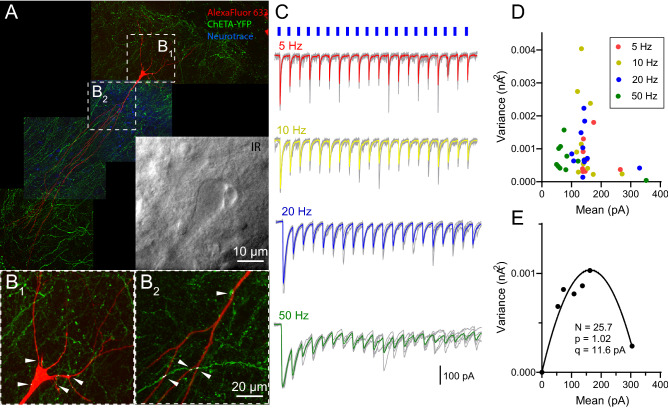
Hippocampal PV-mediated ($\hbox {PV}_{\text{HC}}$) transmission onto a CA1 pyramidal cell (PC). (**A**) Flat-projected confocal image displaying (red) a post-hoc biocytin-filled CA1 PC and (green) ChETA-YFP-containing $\hbox {PV}_{\text{HC}}$ fibers and terminals, counterstained with (blue) Neurotrace 435/455. Inset, Live IR Dodt contrast image of the recorded CA1 PC. (**B1**,**B2**) Expanded images from dotted boxes in A, showing co-localization (white) of (green) ChETA-YFP $\hbox {PV}_{\text{HC}}$ terminals and (red) the recorded CA1 PC. Representative light pulse trains (1 ms duration, 20 pulses (P1–P20), repeated 7 times) at (**C**) 5 Hz, (**D**) 10 Hz, (**E**) 20 Hz, and (**F**) 50 Hz (raw traces, grey; average, colored). (**G**) The variance (V) and peak mean (M) was computed for each pulse in each pulse train in (**C**–**F**). In G, 20 raw VM points are displayed for (red) 5 Hz, (yellow) 10 Hz, (blue) 20 Hz and (green) 50 Hz conditions, with each point representing the M and V of 7 individual IPSCs from P1–P20. (**H**) VM plots from G are binned in 20 pA increments. The VM relationship was fit by a parabolic function (see “Methods” and Fig. 3 legend), enabling estimation of the number of functional release sites (N), release probability (p), and average quantal amplitude (q).

To investigate synaptic dynamics at $\hbox {PV}_{\text{HC}}$ synapses using a similar approach, we injected ChETA-YFP AAV into the CA1 hippocampus of PV-CRE mice. ChETA-YFP fibers and synaptic terminals were detected in all hippocampal layers (Fig. 6A,B), likely indicating synaptic contributions of different PV interneuron subtypes that target different regions of the somatodendritic domain of CA1 PCs, including PV basket cells (PV BC), PV bistratified (PV BiS) cells, chandelier (axoaxonic) cells, and a subset of PV-containing O-LM cells^35,63,64^. Depressing IPSCs were readily evoked from $\hbox {PV}_{\text{HC}}$ synapses from CA1 PCs (Fig. 6C–F). In one cell (Fig. 6A), post-hoc immunocytochemical analysis showed many points of co-localization in perisomatic (Fig. 6B1) and dendritic (Fig. 6B2) regions of the recorded CA1 PC, consistent with contributions of PV-mediated perisomatic and dendritic synapses. V–M analysis on $\hbox {PV}_{\text{HC}}$-mediated IPSCs (Fig. 6G,H) was performed in the same manner as with $\hbox {PV}_{\text{MS-DBB}}$ synapses (Fig. 3F,G). Of 46 recorded CA1 PCs, 36 cells responded to light simulation (78.3%). Light pulse trains at 5–50 Hz elicited large IPSCs with low variance which were depressing upon repetitive stimulation (Fig. 5E), lowering $\hbox {p}_{\text{r}}$ in an activity-dependent manner (Fig. 5F). Consistent with a presynaptic locus, CV increased during 5–50 Hz trains (P1 vs. the average of P11–20, $\hbox {p}<0.01$ for all 5–50 Hz trains, n = 14, 17, 16, 19 for 5 Hz, 10 Hz, 20 Hz, 50 Hz respectively,Wilcoxon matched-pairs signed rank test; Fig. 5G). CV of IPSCs from $\hbox {PV}_{\text{HC}}$ synapses (Fig. 5G) was comparable to CV of IPSCs generated from $\hbox {PV}_{\text{MS-DBB}}$ synapses at all pulses of all frequencies (Two-way ANOVA, $\hbox {p}>0.05$, followed by Sidak’s multiple comparisons test) (Fig. 5C). As in Fig. 5D for $\hbox {PV}_{\text{MS-DBB}}$ synapses, $\hbox {N}_{\text{RP}}$ for $\hbox {PV}_{\text{HC}}$ synapses was calculated to be $32.8 \pm 8.6$ (Fig. 5H), which was not significantly different than the $\hbox {N}_{\text{VM}}$ of $22.8 \pm 5.9$ for $\hbox {PV}_{\text{HC}}$ synapses (Table S2). Access resistance ($\hbox {R}_{\text{a}}$) of recorded PCs was similar with that of SO interneurons ($9.3\pm 0.7$ vs. $10.7 \pm 1.1$ $M\Omega$; n = 19, n = 12; t(29) = 0.59, p = 0.56, unpaired *t* test). Similarly, $\hbox {R}_{\text{a}}$ did not correlate with PPD magnitude, consistent with adequate voltage clamp. VM analysis revealed a higher $\hbox {p}_{\text{max}}$ at $\hbox {PV}_{\text{HC}}$ ($0.87 \pm 0.04$, n = 19) than $\hbox {PV}_{\text{MS-DBB}}$ ($0.64 \pm 0.07$, n = 12) synapses (Table S2; p = 0.01, U(29) = 52, Mann–Whitney test). However, a statistical comparison of $\hbox {N}_{\text{VM}}$ (MS-DBB: $15.4 \pm 4.0$; HC: $22.8 \pm 5.9$, p = 0.35, U(29) = 91) and q (MS-DBB: $36.4 \pm 6.0$ pA; HC: $55.0 \pm 12.0$ pA; p = 0.86, U(29) = 109, Mann–Whitney U) revealed no significance between $\hbox {PV}_{\text{MS-DBB}}$ and $\hbox {PV}_{\text{HC}}$ synapse types (Table S2). In an attempt to directly compare the efficacy of $\hbox {PV}_{\text{HC}}$ to $\hbox {PV}_{\text{MS-DBB}}$ transmission within the same cell population, we also recorded from CA1 SO interneurons. However, we did not observe optogenetically-evoked IPSCs in SO interneurons (0/5), suggesting that SO interneurons are not major targets of axons from local PV interneurons subtypes. This observation is consistent with O-LM cells receiving local GABAergic input predominantly from VIP interneurons^25^.

### $\hbox {PV}_{\text{MS-DBB}}$ synapses are more resistant to STD at gamma frequencies compared to $\hbox {PV}_{\text{HC}}$ synapses

**Figure 7:**
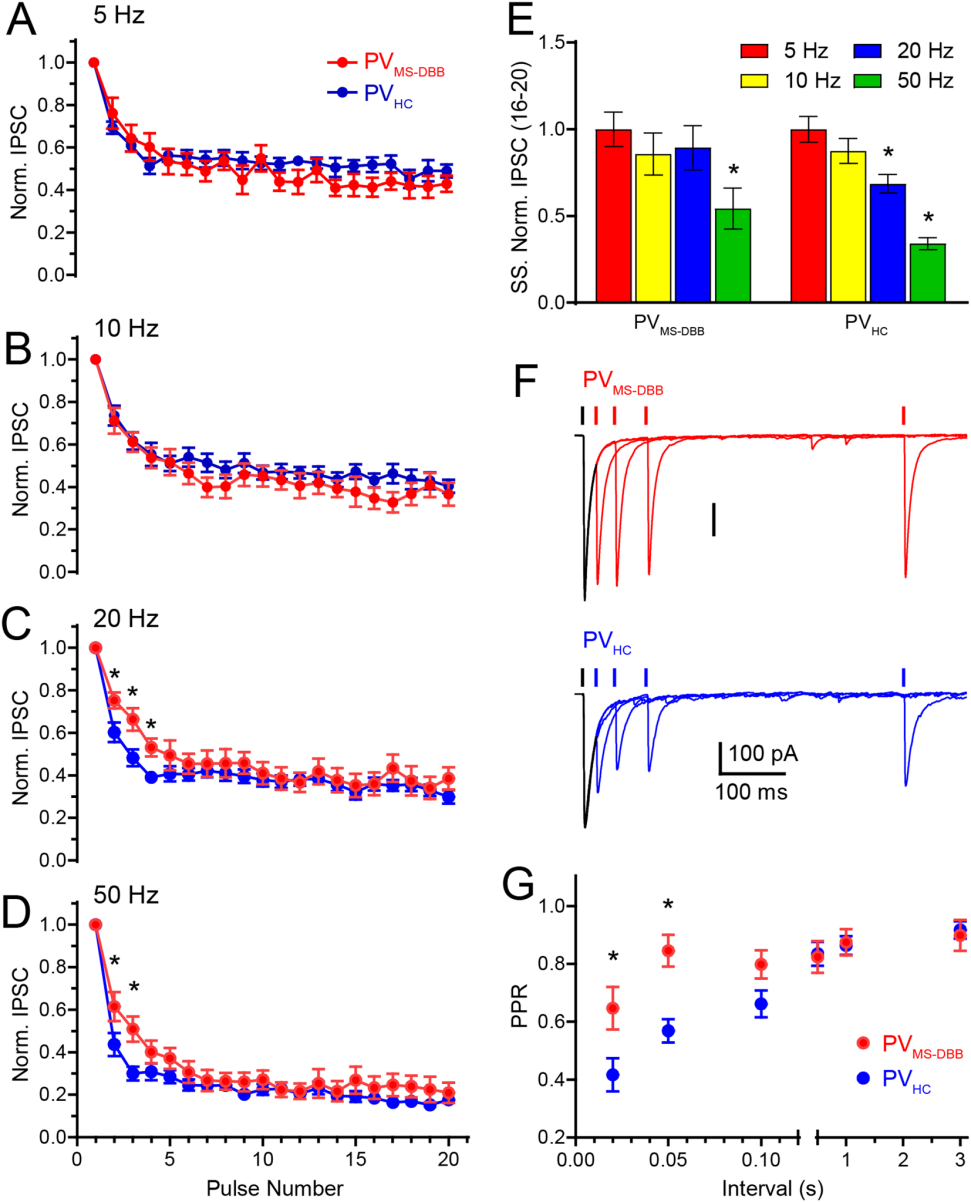
Distinct short-term depression (STD) dynamics at $\hbox {PV}_{\text{MS-DBB}}$ and $\hbox {PV}_{\text{HC}}$ synapses. Normalized optogenetically-evoked IPSC amplitude of P2–P20 relative to P1 amplitude at 5 Hz (**A**), 10 Hz (**B**), 20 Hz (**C**), and 50 Hz (**D**) from $\hbox {PV}_{\text{MS-DBB}}$ (red) and $\hbox {PV}_{\text{HC}}$ (blue) synapse types. * denotes $\hbox {p}<0.05$ (multiple  *t* test). (**E**) Bar graph showing differences in steady state depression (SSD; normalized to average of 16–20th pulses at 5 Hz) of IPSCs at 5–50 Hz frequencies for $\hbox {PV}_{\text{MS-DBB}}$ and $\hbox {PV}_{\text{HC}}$ synapse types. (**F**) Recovery from paired-pulse depression (PPD) for representative $\hbox {PV}_{\text{MS-DBB}}$ (red) and $\hbox {PV}_{\text{HC}}$ (blue) synapse types. Traces are aligned to P1, with a variable delay (20, 50, 100, 500 ms shown) for P2. (**G**) Paired pulse ratio (average P2/average P1) for different intervals (20, 50, 100, 500, 1000 and 3000 ms) from (red) $\hbox {PV}_{\text{MS-DBB}}$ and (blue) $\hbox {PV}_{\text{HC}}$ synapse types. The shortest PPR intervals (20 ms and 50 ms) were significantly different for (red) $\hbox {PV}_{\text{MS-DBB}}$ and (blue) $\hbox {PV}_{\text{HC}}$ synapses (unpaired *t* test, n = 12 and n = 13, respectively, $\hbox {p}<0.05$; denoted by asterisks).

To determine how much STD differed between $\hbox {PV}_{\text{MS-DBB}}$ (n = 12) and $\hbox {PV}_{\text{HC}}$ synapses (n = 19), we normalized P1 amplitude and examined the relative extent of PPD and STD during IPSC trains (Fig. 7A–E). No difference in PPD or STD was observed at 5 Hz (Fig. 7A; $\hbox {p}>0.05$) or 10 Hz (Fig. 7B; $\hbox {p}>0.05$) stimulation (multiple *t* test). However, significant differences emerged at 20 Hz ($\hbox {p}<0.05$ for P2, 3 and 4; Fig. 7C) and 50 Hz ($\hbox {p}<0.001$ for P2 and 3; multiple *t* test, Fig. 7D). At 20 Hz, PPD was larger at $\hbox {PV}_{\text{HC}}$ ($0.60 \pm 0.05$, n = 16) than $\hbox {PV}_{\text{MS-DBB}}$ ($0.75 \pm 0.04$, n = 11) synapses (p = 0.011; Fig. 7C). At 50 Hz, PPD was also larger at $\hbox {PV}_{\text{HC}}$ ($0.44 \pm 0.05$, n = 19) than $\hbox {PV}_{\text{MS-DBB}}$ ($0.62 \pm 0.07$, n = 10) synapses (p = 0.0008, Fig. 7D). Significant differences were also observed for P3–P4 at 20 Hz and P3 at 50 Hz ($\hbox {p}<0.05$) but were not significant later in the train ($\hbox {p}>0.05$ at P5–20, multiple *t* test). These results suggest that, relative to $\hbox {PV}_{\text{HC}}$ synapses, resistance to STD at $\hbox {PV}_{\text{MS-DBB}}$ synapses is greatest during short bursts of 2–4 APs at 20–50 Hz.

To determine whether $\hbox {PV}_{\text{MS-DBB}}$ and $\hbox {PV}_{\text{HC}}$ synapses differed in the extent of STD, we examined steady-state depression (SSD; average of P16–P20) at 10–50 Hz, normalized to the extent of STD at 5 Hz (Fig. 7E). SSD depended on frequency at both $\hbox {PV}_{\text{MS-DBB}}$ and $\hbox {PV}_{\text{HC}}$ synapses, as indicated by a significantly larger SSD at 50 Hz (one-way ANOVA, p = 0.025, $\hbox {p} < 0.0001$, respectively followed by Dunnett’s multiple comparisons test). However, in contrast to $\hbox {PV}_{\text{HC}}$ synapses (p = 0.0017), SSD observed at 20 Hz was not significantly different than 5 Hz at $\hbox {PV}_{\text{MS-DBB}}$ synapses (one way ANOVA, p = 0.853, followed by Dunnett’s multiple comparisons test; Fig. 7E).

This intriguing difference in PPD between $\hbox {PV}_{\text{MS-DBB}}$ and $\hbox {PV}_{\text{HC}}$ synapses led us to investigate the time course of recovery at these synapses. We monitored PPD while varying the interpulse interval at $\hbox {PV}_{\text{MS-DBB}}$ and $\hbox {PV}_{\text{HC}}$ synapses (Fig. 7F,G). Both $\hbox {PV}_{\text{MS-DBB}}$ and $\hbox {PV}_{\text{HC}}$ (Fig. 7F) synapses exhibited PPD at 20–1000 ms intervals ($\hbox {p}<0.05$, one-sample *t* test). PPD was  reduced at $\hbox {PV}_{\text{MS-DBB}}$ synapses for paired stimulation in the gamma range (p = 0.0005 and p = 0.021 for 20 and 50 Hz, Mann Whitney test and unpaired *t* test respectively). Collectively, these observations lead to the conclusion that $\hbox {PV}_{\text{MS-DBB}}$ synapses more effectively transmit short bursts at gamma frequency than $\hbox {PV}_{\text{HC}}$ synapses. Finally, we found that $\hbox {PV}_{\text{MS-DBB}}$-mediated IPSCs exhibited a faster decay time constant ($8.5 \pm 1.3$ ms, n = 12) than $\hbox {PV}_{\text{HC}}$-mediated IPSCs ($13.1 \pm 0.07$ ms; p = 0.023, n = 13, unpaired *t* test). However, there was no significant difference in average IPSC rise-time (10–90%) between $\hbox {PV}_{\text{MS-DBB}}$ ($1.01 \pm 0.09$ ms) and $\hbox {PV}_{\text{HC}}$ ($0.95 \pm 0.10$ ms; p = 0.62, unpaired *t* test) synapses. The similarity in IPSC rise time suggests that electrotonic location alone cannot fully account for differences in IPSC decay time between $\hbox {PV}_{\text{MS-DBB}}$ and $\hbox {PV}_{\text{HC}}$ synapse types. Differences in $\hbox {GABA}_{\text{A}}$ receptor subunit composition and/or synaptic release kinetics may contribute as well.

**Figure 8:**
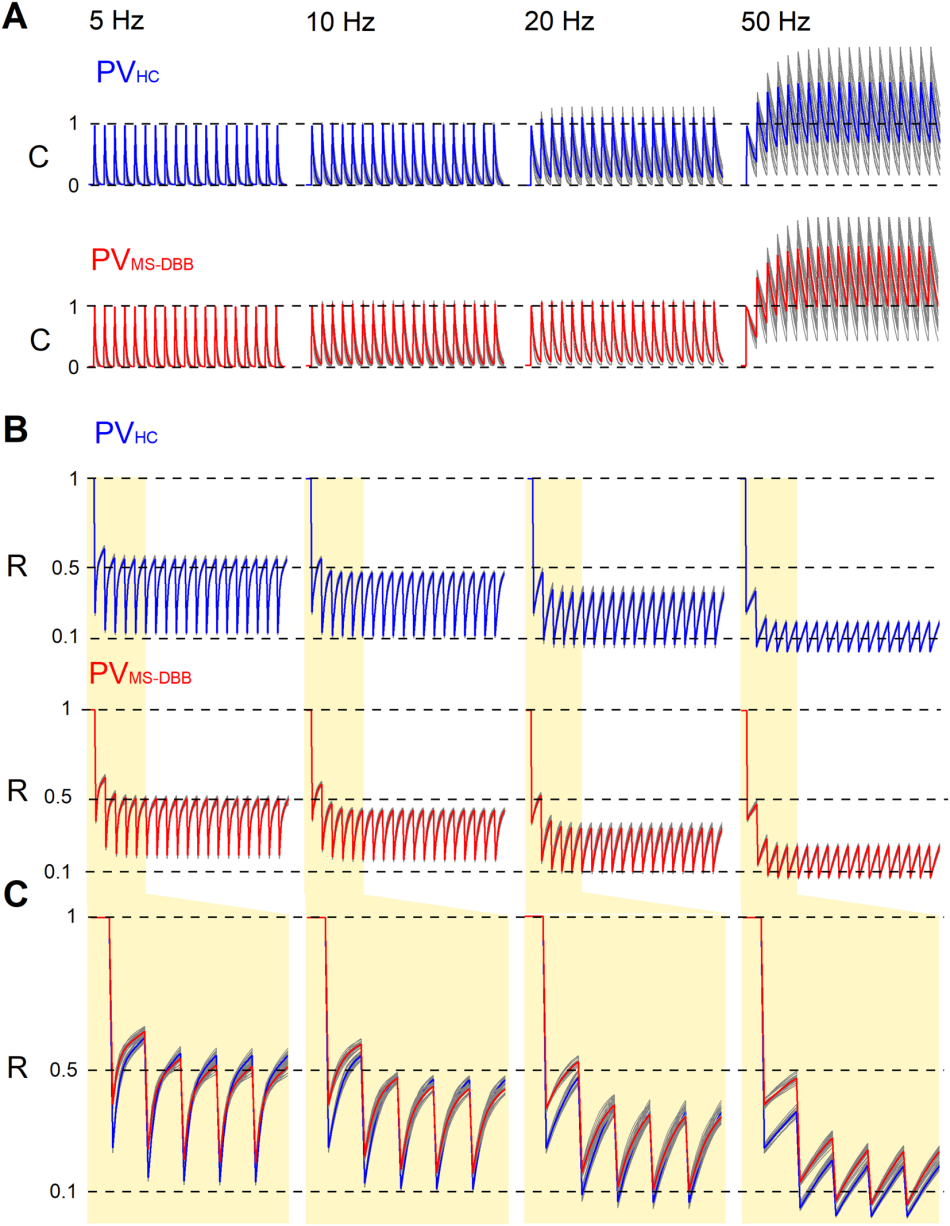
$\hbox {PV}_{\text{MS-DBB}}$ and $\hbox {PV}_{\text{HC}}$ synapse models reproduce differences in STD dynamics. (**A**) Plots of the change in intracellular ($\Delta _{\text{[Ca]}}$ dynamics, C; values 0–1) for $\hbox {PV}_{\text{HC}}$ (blue) and (**B**) $\hbox {PV}_{\text{MS-DBB}}$ (red) synapse models. (**C**) is similar across 5–50 Hz between $\hbox {PV}_{\text{HC}}$ and $\hbox {PV}_{\text{MS-DBB}}$ synapses. (**B**) Fraction of vesicles available for release (R; values 1–0) across 5–50 Hz frequencies for $\hbox {PV}_{\text{HC}}$ (blue) and $\hbox {PV}_{\text{MS-DBB}}$ (red) synapse models. (**C**) The first 5 stimuli in the pulse train from B (yellow region) are shown at expanded scale. At 20 and 50 Hz, activity-dependent accumulation of calcium promotes greater resistance to STD in the $\hbox {PV}_{\text{MS-DBB}}$ synapse model by accelerating calcium-dependent recovery. Grey traces are MCMC simulations; colored traces are the average. Parameters from Table 1 were used for the simulations. See also Figure S6 for comparisons of calcium-dependent recovery rates across calcium concentrations.

### $\hbox {PV}_{\text{MS-DBB}}$ and $\hbox {PV}_{\text{HC}}$ synapse models differ in calcium-dependent recovery from STD

To gain mechanistic insights into underlying activity-dependent differences in STD dynamics between $\hbox {PV}_{\text{MS-DBB}}$ and $\hbox {PV}_{\text{HC}}$ synapses, we combined mathematical modeling with Bayesian parameter estimation techniques, following our earlier work at PV basket cell synapses^44^. After parameters were fit by minimizing the mean square error between the models and the data (see “Methods”), $\hbox {PV}_{\text{HC}}$ and $\hbox {PV}_{\text{MS-DBB}}$ models adequately captured the initial $\hbox {p}_{\text{r}}$, frequency-dependence of STD, and frequency-dependence of SSD across each of the four frequencies examined (5, 10, 20, and 50 Hz; Fig. S5). The kinetic parameters for $\hbox {PV}_{\text{MS-DBB}}$ and $\hbox {PV}_{\text{HC}}$ models are summarized in Table 1. Comparing the $\hbox {PV}_{\text{HC}}$ and $\hbox {PV}_{\text{MS-DBB}}$ model parameters, $\hbox {K}_{\text{r}}$ (which controls the rate of calcium dependent recovery from depression) is approximately 6-fold larger at $\hbox {PV}_{\text{MS-DBB}}$ synapses than $\hbox {PV}_{\text{HC}}$ synapses, suggesting that recovery from depression is more sensitive to calcium accumulation at $\hbox {PV}_{\text{MS-DBB}}$ than $\hbox {PV}_{\text{HC}}$ synapses. The performance of the models across frequencies is shown graphically in Fig. 8. For each synapse type, the time course of the calcium transient, C (Fig. 8A) and the fraction of readily-releasable vesicles available, R (Fig. 8B), are shown^44^. The frequency-dependent summation of C is comparable between $\hbox {PV}_{\text{HC}}$ and $\hbox {PV}_{\text{MS-DBB}}$ synapse types. Consistent with the requirement of calcium-dependent recovery (CDR) to fit STD data from synaptically connected PV BCs-CA1 PC pairs^41,44^, using a constant calcium-independent rate of recovery increased the residual error of the $\hbox {PV}_{\text{HC}}$ model (not shown). Therefore, both $\hbox {PV}_{\text{HC}}$ and $\hbox {PV}_{\text{MS-DBB}}$ models require CDR but differ in the extent that CDR contributes to STD. The lower initial $\hbox {p}_{\text{r}}$ at $\hbox {PV}_{\text{MS-DBB}}$ synapses helps to protect R from depletion (Fig. 8C). Upon repetitive stimulation at 5 Hz, $\hbox {PV}_{\text{HC}}$ synapses have a higher calcium-independent rate of recovery ($\hbox {k}_{\text{min}}$) than $\hbox {PV}_{\text{MS-DBB}}$ synapses, partially offsetting the enhanced $\hbox {p}_{\text{r}}$ and profound depletion of R at $\hbox {PV}_{\text{HC}}$ synapses. However, at 20 and 50 Hz frequencies, the larger $\hbox {K}_{\text{r}}$ of $\hbox {PV}_{\text{MS-DBB}}$ synapses enables $\hbox {PV}_{\text{MS-DBB}}$ synapses to respond to increased calcium accumulation with an enhanced CDR rate. In contrast, $\hbox {K}_{\text{r}}$ is readily saturated at $\hbox {PV}_{\text{HC}}$ synapses and becomes relatively insensitive to additional calcium accumulation at 20 and 50 Hz, worsening depletion of R at these frequencies relative to $\hbox {PV}_{\text{MS-DBB}}$ synapses (Fig. S6). These differences may partly explain experimental differences between $\hbox {PV}_{\text{HC}}$ and $\hbox {PV}_{\text{MS-DBB}}$ synapses observed in the initial stages of STD at 20 Hz (Fig. 7C) and 50 Hz (Fig. 7D), and recovery from PPD (Fig. 7G).

## Discussion

In this study, we used patch-clamp electrophysiology and optogenetics to investigate the cellular and synaptic properties of $\hbox {PV}_{\text{MS-DBB}}$ neurons. $\hbox {PV}_{\text{MS-DBB}}$ neurons exhibited a fast spiking phenotype that was distinct from $\hbox {SOM}_{\text{MS-DBB}}$ and $\hbox {ChAT}_{\text{MS-DBB}}$ cells. To examine frequency-dependent synaptic dynamics at $\hbox {PV}_{\text{MS-DBB}}$ synapses, we optogenetically investigated $\hbox {PV}_{\text{MS-DBB}}$-transmission across a range of physiological firing frequencies, inducing IPSCs in CA1 SO interneurons. Using similar stimulation protocols, we also examined the frequency-dependence of  $\hbox {PV}_{\text{HC}}$-mediated transmission onto CA1 pyramidal cells. $\hbox {PV}_{\text{MS-DBB}}$ synapses exhibited greater resistance to STD during high frequency gamma bursts than at $\hbox {PV}_{\text{HC}}$ synapses. Mathematical synapse models demonstrated that resistance to STD at $\hbox {PV}_{\text{MS-DBB}}$ synapses can be explained by greater sensitivity to calcium accumulation, accelerating calcium-dependent recovery from depression.

Previous studies in rat MS-DBB classified neuronal types as slow-firing, burst-firing or fast-firing^55,65,66^. Similar to fast-spiking neurons in mouse MS-DBB^67^ and basal forebrain^68^, $\hbox {PV}_{\text{MS-DBB}}$ neurons had low $\hbox {R}_{\text{in}}$, large $\hbox {C}_{\text{m}}$, low sag ratio, and a narrow AP half-width. These properties were distinct from $\hbox {SOM}_{\text{MS-DBB}}$ and $\hbox {ChAT}_{\text{MS-DBB}}$ neurons (Figs. 1, 2; Table S1). Consistent with rat MS-DBB GABAergic neurons^31,57,66,67^, sag was common to both $\hbox {SOM}_{\text{MS-DBB}}$ and $\hbox {PV}_{\text{MS-DBB}}$ neurons. The combination of low $\hbox {R}_{\text{in}}$, large $\hbox {C}_{\text{m}}$, and brief AP half-width allows $\hbox {PV}_{\text{MS-DBB}}$ neurons to tolerate larger currents and sustain higher frequency firing than $\hbox {SOM}_{\text{MS-DBB}}$ neurons (Fig. 1A), which likely contribute to the firing properties of $\hbox {PV}_{\text{MS-DBB}}$ neurons in vivo^30,31^ and permit faithful propagation of information to the hippocampus^5^. Despite the lower $\hbox {R}_{\text{in}}$ of $\hbox {PV}_{\text{MS-DBB}}$ neurons, the sag ratio between $\hbox {SOM}_{\text{MS-DBB}}$ and $\hbox {PV}_{\text{MS-DBB}}$ neurons was similar (Table S1), suggesting a higher HCN channel density in $\hbox {PV}_{\text{MS-DBB}}$ than $\hbox {SOM}_{\text{MS-DBB}}$ neurons. Therefore, Kv3.1^69^ and HCN1/2^31^ expression are likely key molecular determinants of fast-spiking and pacemaking^31^ ability of $\hbox {PV}_{\text{MS-DBB}}$ neurons. Consistent with the wider AP half-width, $\hbox {SOM}_{\text{MS-DBB}}$ neurons may form a fast-spiking, accommodating class of MS-DBB neurons^65^ that contain conductances involved in spike accommodation that are not present in $\hbox {PV}_{\text{MS-DBB}}$ neurons^70^. Although $\hbox {PV}_{\text{MS-DBB}}$ and $\hbox {ChAT}_{\text{MS-DBB}}$ populations are considered mutually exclusive (Fig. S1), anti-ChAT labeling (not shown) suggested some overlap between ChAT and SOM populations, raising the possibility that $\hbox {SOM}_{\text{MS-DBB}}$ neurons also co-release ACh. Future single cell transcriptomics studies will inform the diverse classes of MS-DBB subtypes suggested here. In addition to electrophysiological differences, these neurochemically distinct cell populations were differentially localized within MS-DBB. Consistent with studies in rat MS-DBB^18,19,45–47,49^, $\hbox {PV}_{\text{MS-DBB}}$ neurons were localized to the MS midline, whereas $\hbox {SOM}_{\text{MS-DBB}}$ and $\hbox {ChAT}_{\text{MS-DBB}}$ neurons were more diffusely localized to MS-DBB and LSN (Fig. 1).

The hippocampal theta rhythm emerges from enhanced functional connectivity between MS-DBB and hippocampus^22,71^. $\hbox {PV}_{\text{MS-DBB}}$ neurons are active participants in hippocampal theta oscillations^1,72–75^, which are important for sensory processing^5^, memory retrieval^7^, and memory consolidation^8^. A subset of $\hbox {PV}_{\text{MS-DBB}}$ neurons lead hippocampal neurons in the theta rhythm, implying that $\hbox {PV}_{\text{MS-DBB}}$ neurons pace hippocampal theta oscillations^2,3,73^. $\hbox {PV}_{\text{MS-DBB}}$ neurons can effectively transmit gamma oscillations to cortex^76^ and hippocampus^59^. Several neurochemically distinct subtypes of MS-DBB neurons project to the hippocampus, which currently include $\hbox {PV}_{\text{MS-DBB}}$ neurons^15,19^, $\hbox {ChAT}_{\text{MS-DBB}}$ neurons (recently found to co-release ACh and GABA)^77^), and glutamatergic MS-DBB neurons^78–80^. Previous work using minimal electrical stimulation of MS-DBB GABAergic fibers, in the presence of glutamate receptor blockers, demonstrated resistance of IPSCs to PPD and STD at 10 Hz stimulation in CA1 O-LM cells. By contrast, robust PPD and STD from local afferents, likely from VIP-positive axons, were observed in CA1 O-LM cells^25^. A difference in quantal amplitude was also observed between $\hbox {PV}_{\text{MS-DBB}}$ and $\hbox {PV}_{\text{HC}}$ synapse types, suggesting differential electrotonic filtering, $\hbox {GABA}_{\text{A}}$ receptor number, and/or single channel $\hbox {GABA}_{\text{A}}$ conductance^25^. Here, we investigated $\hbox {PV}_{\text{MS-DBB}}$ transmission onto CA1 SO cells using a CRE-dependent AAV containing the fast kinetic channelrhodopsin ChETA for fast optogenetic control suitable for PV-mediated transmission^40^. Compared to $\hbox {PV}_{\text{HC}}$ synapses, during the initial pulses of the optogenetic pulse train, $\hbox {PV}_{\text{MS-DBB}}$ synapses exhibited less STD of optically evoked IPSCs at intervals at the start of 20–50 Hz pulse trains (Fig. 7C,D). Recovery from PPD was also faster at $\hbox {PV}_{\text{MS-DBB}}$ than $\hbox {PV}_{\text{HC}}$ synapses (Fig. 7F–G).

At the ultrastructural level, the size of MS-DBB GABAergic terminals is larger than local hippocampal GABAergic terminals^24^, which may relate to differences in intracellular calcium dynamics and/or vesicular storage capacity. Although the difference in STD between $\hbox {PV}_{\text{MS-DBB}}$ and $\hbox {PV}_{\text{HC}}$ synapses may be partly accounted for by the lower initial $\hbox {p}_{\text{r}}$ at $\hbox {PV}_{\text{MS-DBB}}$ synapses, a larger readily releasable pool at $\hbox {PV}_{\text{MS-DBB}}$ synapses may also contribute to differences in the onset of STD between $\hbox {PV}_{\text{MS-DBB}}$ and $\hbox {PV}_{\text{HC}}$ synapses. Our experimental and modeling results also suggest that there are differences in calcium binding proteins mediating the activity-dependence of CDR. Possible molecular determinants include the synaptic vesicle priming protein Munc13-1^81^, the calcium sensor synaptotagmin 7^82^, and others^83^. Since the postsynaptic neuron types were different for $\hbox {PV}_{\text{MS-DBB}}$ and $\hbox {PV}_{\text{HC}}$ synapses, we cannot rule out target-specific effects of STD. Ideally, target-specificity could be tested by examining a common interneuron target between $\hbox {PV}_{\text{MS-DBB}}$ synapses and $\hbox {PV}_{\text{HC}}$ synapses. Although we did not perform an exhaustive investigation, we were unsuccessful in optogenetically evoking $\hbox {PV}_{\text{HC}}$-mediated transmission onto stratum oriens interneurons, consistent with the paucity of published studies on synaptic connections from $\hbox {PV}_{\text{HC}}$ to O-LM neurons. Other $\hbox {PV}_{\text{MS-DBB}}$ targets in the hippocampus, such as CCK interneurons in stratum radiatum^16^, also do not receive significant synaptic input from $\hbox {PV}_{\text{HC}}$ synapses^84^. However, $\hbox {PV}_{\text{HC}}$ neurons receive input from both $\hbox {PV}_{\text{MS-DBB}}$^12,16^ and themselves^85^. If STD resistance generalizes to other postsynaptic targets such as PV basket cells and axoaxonic cells, which have briefer membrane time constant^86^, there may be synergism between $\hbox {PV}_{\text{MS-DBB}}$ and local $\hbox {PV}_{\text{HC}}$ microcircuits in the transmission and/or amplification of gamma oscillations to the hippocampus.

$\hbox {PV}_{\text{MS-DBB}}$ neurons are observed to fire short bursts of APs at gamma frequency, nested in a theta rhythm^1,11^. In accordance with in vivo observations^59,76^, we demonstrate that this range of firing frequencies is conducive to $\hbox {PV}_{\text{MS-DBB}}$ transmission at gamma frequencies, supported both by experimental and modeling results. However, naturalistic patterns of $\hbox {PV}_{\text{MS-DBB}}$-mediated IPSPs are likely to impact postsynaptic target cells differently depending on their membrane time constant and resonance properties^86,87^. In multi-compartmental models of O-LM cells, IPSPs readily generate theta resonance in part through hyperpolarization-induced recruitment of HCN channels^86,88,89^, supporting the idea that rhythmic disinhibition via phasic $\hbox {PV}_{\text{MS-DBB}}$ transmission contributes to theta rhythm generation^90^. Due to the fact that O-LM cells have relatively long membrane time constants^35^, bursts of IPSPs at gamma frequency are likely to be integrated, with a burst of depression-resistant IPSPs at gamma frequency to be effective in driving hyperpolarization-induced activation of HCN channels, rebound spikes, and inducing synchronous disinhibition of hippocampal principal cells^19^. Future modeling studies that enable independent manipulation of pre- and postsynaptic parameters will enable the physiological consequences of $\hbox {PV}_{\text{MS-DBB}}$ transmission onto O-LM cells to be fully understood. Presynaptic and/or postsynaptic cholinergic neuromodulation^41,91,92^ may further tune $\hbox {PV}_{\text{MS-DBB}}$-O-LM transmission, possibly interacting with presynaptic $\hbox {GABA}_{\text{B}}$ receptors^27^ to protect the readily-releasable vesicle pool from excessive depletion during gamma frequency bursts. However, it is likely that PV basket cells, PV bistratified cells, and chandelier (axoaxonic) cells, which have short membrane time constants and exhibit gamma resonance^86^, may be more effective in generating discrete gamma frequency IPSPs from $\hbox {PV}_{\text{MS-DBB}}$ synapses than O-LM cells. These cell type-specific differences in integrative properties may account, at least in part, for the differential phase locking of these cell types to gamma oscillations^93^. Finally, differences in short-term plasticity may exist across $\hbox {PV}_{\text{MS-DBB}}$ postsynaptic hippocampal targets. Recent studies have identified a specific subtype of $\hbox {PV}_{\text{MS-DBB}}$ neurons, the Teevra cells, that preferentially innervate axoaxonic cells in CA3^12^. Although we anticipated finding other stratum oriens subtypes among the 12 recorded neurons that responded to optogenetic $\hbox {PV}_{\text{MS-DBB}}$ stimulation, only 2 O-LM cells were anatomically recovered in this population, precluding an investigation of postsynaptic target differences. Nevertheless, this observation is in accord with the idea that SOM-containing cells in stratum oriens are preferred targets^16^. In a more recent study, PV interneurons within stratum oriens were found to be common targets of $\hbox {PV}_{\text{MS-DBB}}$ synapses^13^. Some somatostatin (SOM)-containing dendrites were likely missed due to the poor localization of SOM to dendrites^13^; however, a subset of OLM cells contain PV^64^.

Despite similar IPSC rise times, the IPSC decay was faster at $\hbox {PV}_{\text{MS-DBB}}$- than $\hbox {PV}_{\text{HC}}$-mediated synapses. Local $\hbox {PV}_{\text{HC}}$ neurons are comprised of many anatomically distinct subtypes, including PV basket cells (BC), bistratified cells (BiS), and chandelier (axoaxonic) cells^54^, and a subset of O-LM cells have been shown to contain PV^64^. The near-simultaneous optogenetic activation of perisomatic and dendritic IPSCs from these cell types may interact to prolong the decay of the composite IPSC. A future study using an intersectional optogenetic approach could allow optogenetic activation of specific postsynaptic cell types^94^. However, in paired recordings from BC and BiS cells, IPSC decay times do not substantially differ^63^. Therefore, it is possible that synaptically localized $\hbox {GABA}_{\text{A}}$ activated by $\hbox {PV}_{\text{MS-DBB}}$ and $\hbox {PV}_{\text{HC}}$ axons differ in subunit composition. $\hbox {GABA}_{\text{A}}$ receptors synaptically activated by VIP-positive or $\hbox {PV}_{\text{MS-DBB}}$ afferents on O-LM cells may also differ in $\hbox {GABA}_{\text{A}}$ receptor subunit composition^95^. Alternatively, molecular differences between $\hbox {PV}_{\text{MS-DBB}}$ and $\hbox {PV}_{\text{HC}}$ synapse types, supported by our STD results here, may give rise to subtle differences in release kinetics that could explain the differences in average IPSC decay.

This is the first study to directly examine $\hbox {PV}_{\text{MS-DBB}}$ transmission in isolation of other MS-DBB subtypes known to contribute to septohippocampal GABAergic transmission. Therefore, compared to previous studies of MS-DBB GABAergic transmission^7,25,27^, some differences between our study and previous studies that use differing methodology are expected. We directly stimulated ChETA-YFP channels localized to $\hbox {PV}_{\text{MS-DBB}}$ axons and synapses within an acute hippocampal slice preparation, which enabled us to investigate $\hbox {PV}_{\text{MS-DBB}}$-mediated transmission without the need for a septohippocampal slice preparation. Although optogenetics continues to be an exciting technology to investigate neurochemically distinct projection neurons, there are caveats that have been recognized as the field has evolved^96^. Depending on the synapse type, short-term dynamics using optogenetic stimulation may differ substantially from electrical stimulation^97^. Even subtle variations of the optogenetic technique, such as the AAV serotype used and the subcellular compartment stimulated, can influence short-term plasticity dynamics^97^. In our experiments, we used the rapidly deactivating variant ChETA-YFP, which is superior to first-generation channelrhodopsins in enabling high frequency firing in PV neurons^40^. As observed for the Purkinje cell-deep cerebellar nucleus synapse, optogenetic activation of GABAergic synapses may be more comparable to electrical stimulation, due to high expression of fast potassium channels and enhanced calcium buffering capacity, enabling rapid repolarization of presynaptic boutons^97^. Although we cannot rule out that entry of calcium through ChETA channels influenced STD dynamics, for $\hbox {PV}_{\text{HC}}$ synapses, optogenetically induced STD at $\hbox {PV}_{\text{HC}}$ synapses shown here (Fig. 6) was similar to our earlier work in which single synaptically connected $\hbox {PV}_{\text{HC}}$ axons were electrically stimulated^41,44^. Assuming comparable ChETA-YFP kinetics and densities, our results likely reflect true differences in STD between $\hbox {PV}_{\text{MS-DBB}}$ and $\hbox {PV}_{\text{HC}}$ synapses, which relate to differences in ultrastructure^24^, as well as differences in the type and/or composition of calcium binding proteins mediating recovery from STD. Consistent with this idea, our mathematical models of $\hbox {PV}_{\text{MS-DBB}}$ and $\hbox {PV}_{\text{HC}}$-mediated transmission, in combination with Bayesian parameter estimation techniques, enabled us to quantify the error in estimated parameters, ensuring that it was small enough to make each parameter meaningful. However, any model is a simplification of the underlying biological processes, and it is likely that many distinct kinetic processes are combined within each parameter that are not possible to distinguish here. This appears to be especially true with our measures of calcium decay time course, which is likely to involve not just the calcium transient itself, but numerous “residual” calcium binding proteins that influence both facilitation and depression mechanisms. Moreover, we noted that $\hbox {K}_{\text{r}}$ and $\tau _{\text{Ca}}$ are interdepdendent at both synapse types, which limited our ability to fully constrain these parameters. Nevertheless, our major conclusion, that $\hbox {PV}_{\text{MS-DBB}}$ synapses are resistant to STD, extends earlier observations^25,27^. Beyond the novel experimental results reported here, our initial, basic mathematical description of $\hbox {PV}_{\text{MS-DBB}}$-mediated transmission, which could be refined in future studies, is simple enough to be applied to testing hypotheses in network models of MS-DBB-driven hippocampal gamma and theta rhythms^98,99^.

Finally, in addition to their major roles in hippocampal processing in the normal brain, dysfunction of $\hbox {PV}_{\text{MS-DBB}}$ synapses may contribute to hippocampal dysfunction in disease states. In an Alzheimer’s disease (AD) mouse model, degeneration of the GABAergic septohippocampal pathway is associated with reduction in the magnitude of theta and gamma rhythms^100^. However, stimulation of $\hbox {PV}_{\text{MS-DBB}}$ synapses at gamma frequency rescued cognitive impairment in this same mouse model^59^. Therefore, our modeling of the synaptic dynamics of $\hbox {PV}_{\text{MS-DBB}}$-mediated transmission will enable $\hbox {PV}_{\text{MS-DBB}}$ circuit elements to be built into network models, integrating synaptic dynamics with network oscillations in neural network models of normal and disease states.

## Supplementary information

Supplementary Information.

## Data Availability

The original data and is available from J.J.L. upon request. Matlab code is available from H.H. and E.S.
