# Supplementary material for: Septohippocampal transmission from parvalbumin-positive neurons features rapid recovery from synaptic depression
Source: Sci Rep. 2021 Jan 22;11:2117. doi: 10.1038/s41598-020-80245-w (PMC7822967; doi:10.1038/s41598-020-80245-w)
Supplement: Supplementary file 1 — Supplementary Information. [file 41598_2020_80245_MOESM1_ESM.pdf]

Supplementary Materials

Septohippocampal transmission from parvalbumin-positive neurons features rapid recovery from synaptic depression

Yi, F., Garrett, T., Deisseroth, K., Haario, H., Stone, E., and Lawrence, J. J.

Table S1. Passive and active membrane properties of $PV_{MS\text{-}DBB}$, $SOM_{MS\text{-}DBB}$, and $ChAT_{MS\text{-}DBB}$ neurons.
*compared to $SOM_{MS\text{-}DBB}$ neurons p<0.05.^as compared to $ChAT_{MS\text{-}DBB}$ p<0.05.

Property	$PV_{MS\text{-}DBB}$ (n=9)	$SOM_{MS\text{-}DBB}$ (n=9)	$ChAT_{MS\text{-}DBB}$ (n=10)
R_{in} ($M\Omega$)	158.0 ± 26.0 *^	308.7 ± 46.1	352.0 ± 16.7
1st AP Half Width (ms)	0.45 ± 0.03^	0.63 ± 0.11^	1.20 ± 0.08
1st AP Height (mV)	44.9 ± 2.6	51.6 ± 2.3	44.8 ± 2.6
C_m (pF)	116.7 ± 22.7*^	66.9 ± 11.2	58.6 ± 5.5
Sag ratio (SS/peak) at -200 pA	0.73 ± 0.05	0.82 ± 0.04	0.96 ± 0.01
rebound spikes (# cells)	5/9 (55.5%)	7/9 (77.7%)	1/10 (10%)
τ_m (ms)	15.7 ± 2.7	18.5 ± 3.1	20.3 ± 1.9
Frequency at 100 pA (Hz)	14.1 ± 3.9*	38.7 ± 9.6^	4.1 ± 0.7
Frequency at 400 pA (Hz)	79.4 ± 18.7	141.9 ± 27.1^	1.6 ± 0.2 (n=5)
F-I slope 0-400 pA (Hz/pA)	0.1989	0.3708	0.0022
I_{hold} (pA)	-68.4 ± 18.3*	-16.5 ± 6.7	-32.9 ± 13.7
resting membrane potential	-55.7 ± 2.0	-58.1 ± 2.7	-52.0 ± 2.3 (n=8)

Table S2. Quantal parameters for $PV_{MS\text{-}DBB}$ and PV_{HC} synapses.
*denotes significant difference compared to PV_{HC} neurons. p = 0.011, Mann Whitney test.

Quantal parameters	$PV_{MS\text{-}DBB}$	PV_{HC}
q (pA)	36.4 ± 6.0 (n=12)	55.0 ± 12.0 (n=19)
p_{max}	0.64 ± 0.07 (n=12)*	0.87 ± 0.04 (n=19)
N_{VM}	15.4 ± 4.0 (n=12)	23.9 ± 6.6 (n=19)
N_{AC}	17.8 ± 10.8 (n=4)	14.5 ± 2.3 (n=11)
N_{RP}	17.2 ± 2.3 (n=12)	32.8 ± 8.6 (n=19)

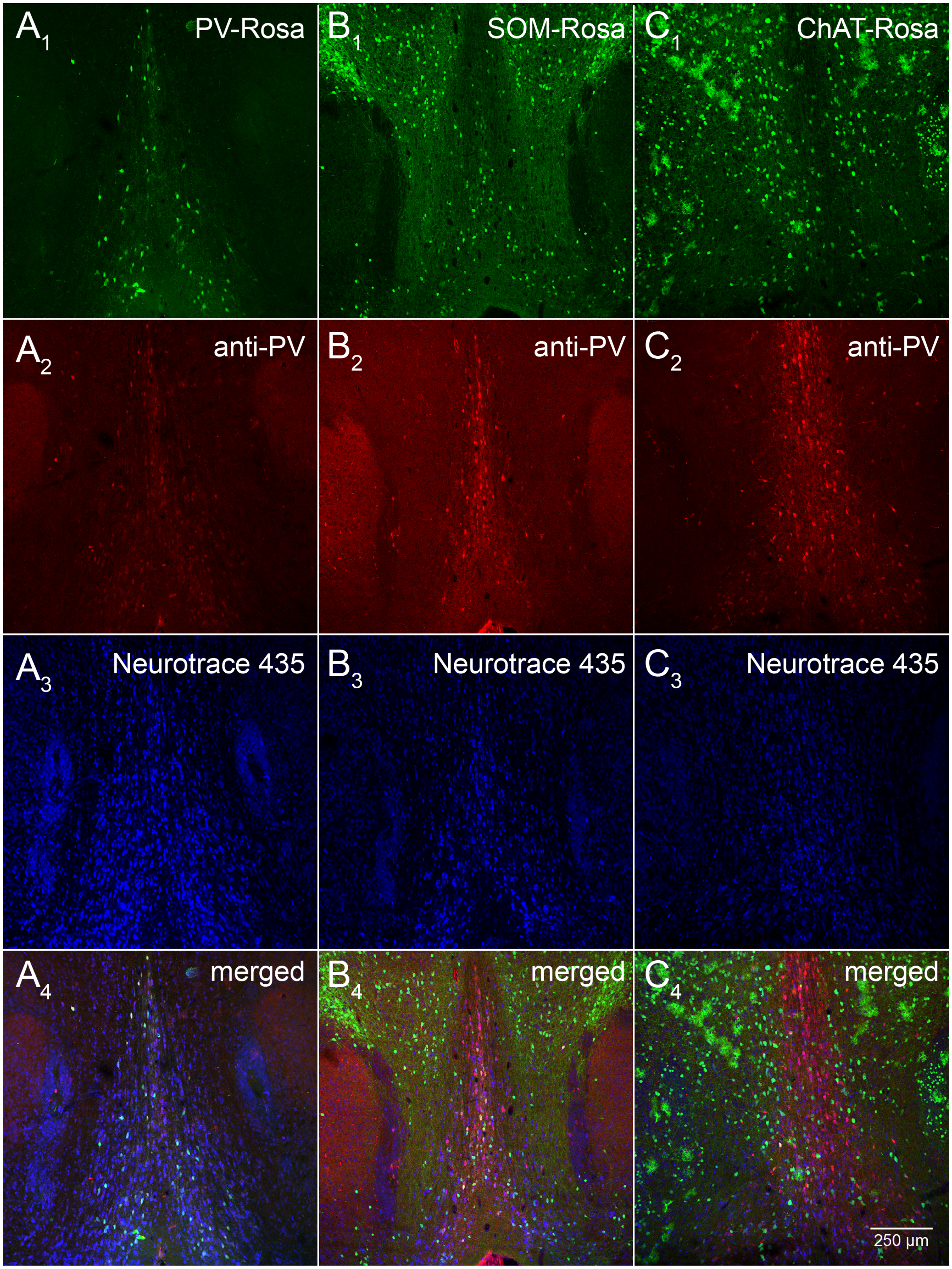

Figure S1. $PV_{MS\text{-}DBB}$, $SOM_{MS\text{-}DBB}$ and $ChAT_{MS\text{-}DBB}$ neurons have different distributions within MS-DBB. (A) YFP-positive neurons (green) from (A) PV-CRE:Rosa26YFP, (B) SOM-CRE:Rosa26YFP, or (C) ChAT-CRE:Rosa26YFP mice were stained with an anti-PV antibody (A2,B2,C2) and counterstained with Neurotrace 435/455 (A3,B3,C3), respectively. Channels were merged in A4,B4, and C4, respectively.

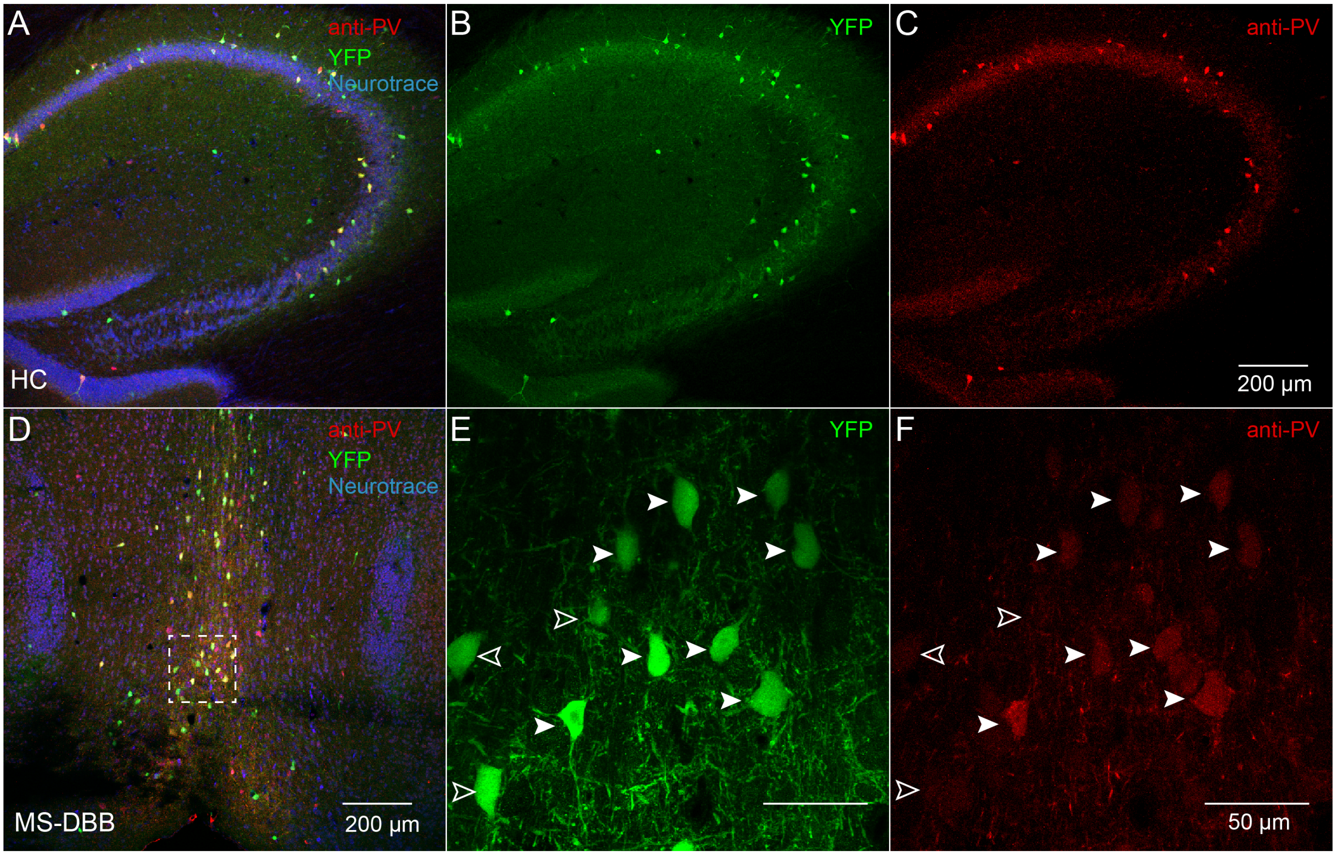

Figure S2. Specificity of CRE expression in the MS-DBB of PV-CRE:Rosa26YFP mice.(A) A representative flat-projected confocal stack from the hippocampus. Merged channels of YFP-positive neurons intensified with an anti-GFP antibody (green), anti-PV labeling (red), and Neurotrace 435/455 (blue), with YFP and anti-PV channels shown separately in B and C, respectively. (D) A representative flat-projected confocal stack of MS-DBB from a PV-CRE:Rosa26YFP mice. Expanded regions from dotted box in D are shown in E and F, demonstrating clear localization of YFP and anti-PV in most $PV_{MS\text{-}DBB}$ neurons (solid arrows). However, in some YFP neurons, anti-PV was not clearly detected (open arrows).

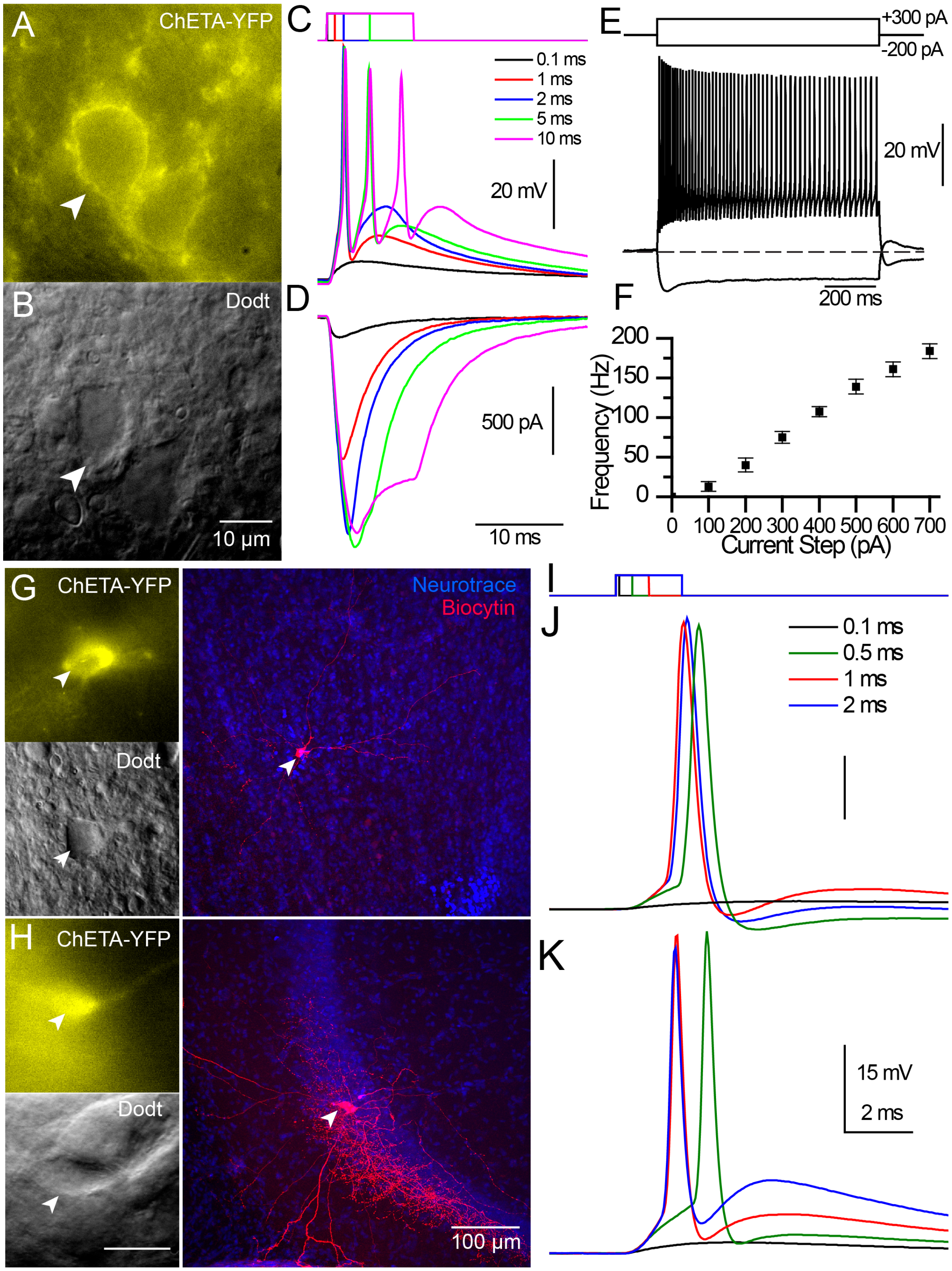

Figure S3. Optogenetic stimulation of $PV_{MS\text{-}DBB}$ and PV_{HC} neurons. (A) Live 505 nm fluorescence and (B) IR-Dodt images of a $PV_{MS\text{-}DBB}$ neuron expressing ChETA-YFP. (C) Current clamp and (D) voltage clamp responses to light pulses of progressively longer durations (0.1-10 ms) were delivered to the recorded cell (arrow in A,B). (E) Voltage responses to the same $PV_{MS\text{-}DBB}$ neuron (as in A-C) to 1 s long, -200 pA and +300 pA current steps from -60 mV (dashed line), demonstrating a fast-spiking phenotype and a hyperpolarization-induced sag. (F) AP frequency-current relationship for 6 ChETA-YFP-expressing $PV_{MS\text{-}DBB}$ neurons. For a 1 ms light pulse, onset was 0.96 ms and offset was 3.74 ms. (I) Light pulses (0.1, 0.5, 1, and 2 ms at 30% power) were given to (J) $PV_{MS\text{-}DBB}$ or (K) PV_{HC} neurons, respectively.

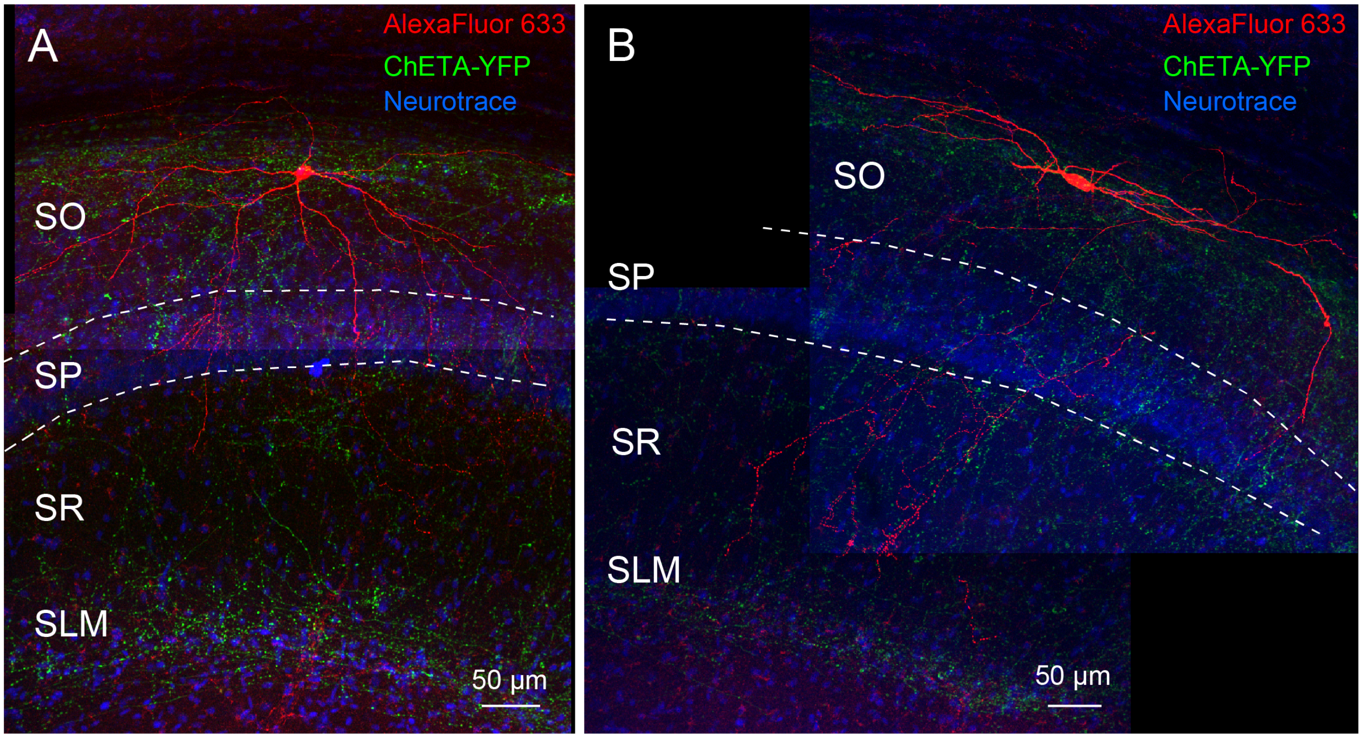

Figure S4. Recorded hippocampal CA1 stratum oriens intereurons have axonal arborizations consistent with that of O-LM neurons. (A-B) More complete images of neurons from Figure 3Ab and Figure 4Ab illustrating axons descending into stratum lacunosum moleculare (SLM). Abbreviations: stratum oriens (SO), stratum pyramidale (SP), stratum radiatum (SR). The Neurotrace 435/455 channel has been included to delineate hippocampal layers.

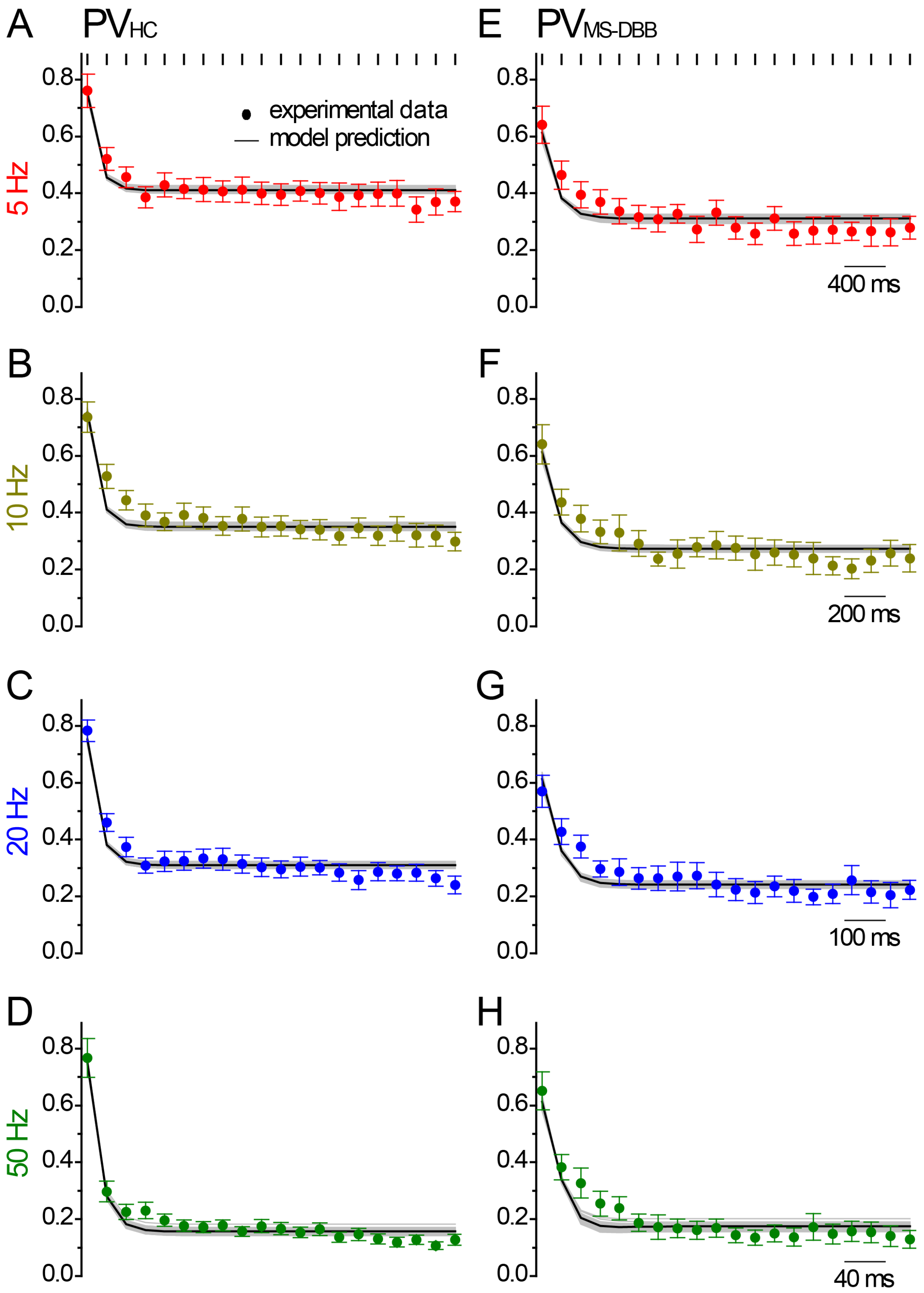

Figure S5. Short-term plasticity (STP) model fits to PV_{HC} and $PV_{MS\text{-}DBB}$ experimental data. STP model fits for 5-50 Hz frequencies for (A-D) and (E-H) PV_{HC} experimental data. Closed symbols indicate replotted data from Fig. 5B and Fig. 5F. Grey lines indicate 100 individual MCMC chains; black lines indicate the average.

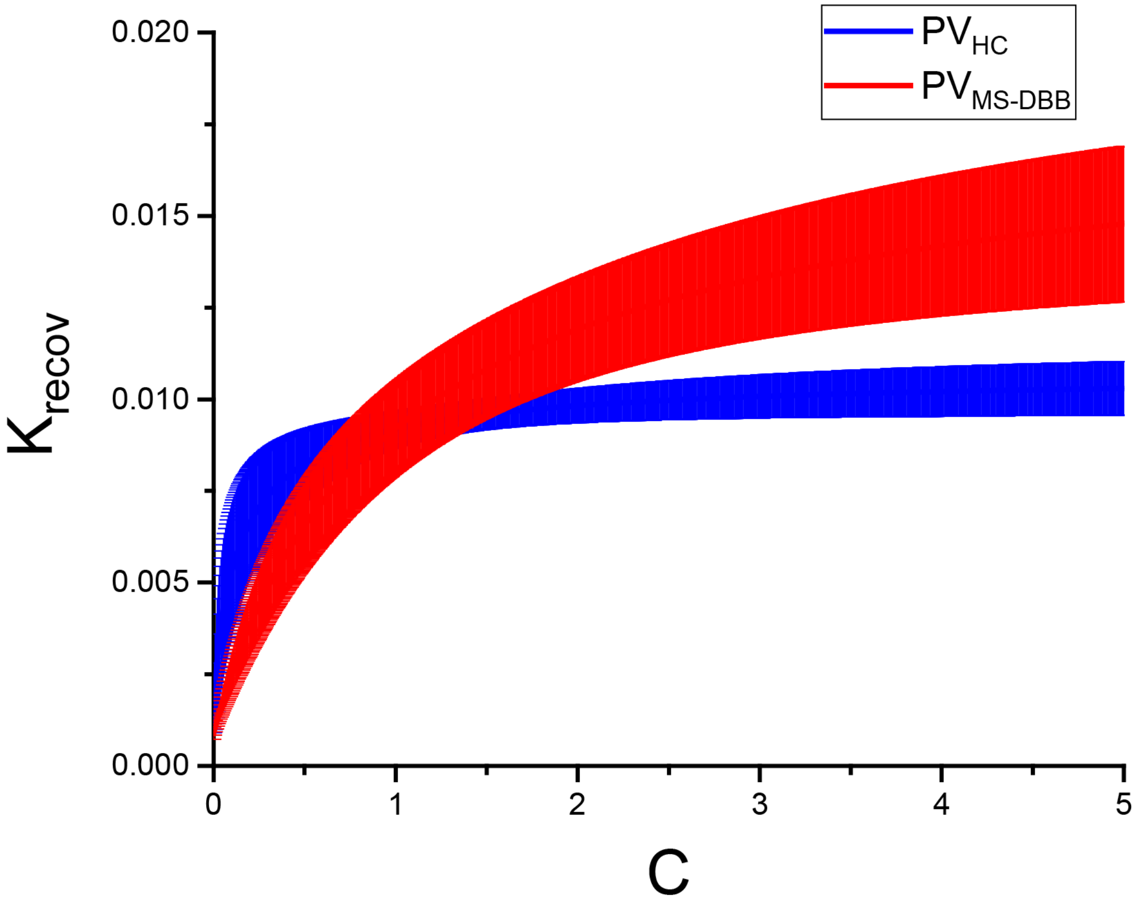

Figure S6. Differences in calcium-dependent recovery rates between $PV_{MS\text{-}DBB}$ and PV_{HC} models. Recovery rate (K_{recov}) as a function of peak internal calcium concentration for PV_{HC} (blue) and $PV_{MS\text{-}DBB}$ (red) synapse models. Red and blue lines represent the mean and standard deviation of 1000 MCMC trials.
